# Structural basis for CRMP2-induced axonal microtubule formation

**DOI:** 10.1038/s41598-017-11031-4

**Published:** 2017-09-06

**Authors:** Shinsuke Niwa, Fumio Nakamura, Yuri Tomabechi, Mari Aoki, Hideki Shigematsu, Takashi Matsumoto, Atsushi Yamagata, Shuya Fukai, Nobutaka Hirokawa, Yoshio Goshima, Mikako Shirouzu, Ryo Nitta

**Affiliations:** 1 Frontier Research Institute for Interdisciplinary Sciences and Department of Life Sciences, Tohoku University, Aoba-ku, Sendai, 980-8578 Japan; 2 Department of Molecular Pharmacology and Neurobiology, Yokohama City University Graduate School of Medicine, Kanazawa-ku, Yokohama, 236-0004 Japan; 3 Department of Biochemistry, Tokyo Women’s Medical University, Shinjuku-ku, Tokyo, 162-8666 Japan; 4 RIKEN Center for Life Science Technologies, Tsurumi-ku, Yokohama, 230-0045 Japan; 5 Application Laboratories, Rigaku Corporation, 3-9-12 Matsubara-Cho, Akishima, Tokyo, 196-8666 Japan; 6 Structural Biology Laboratory, Life Science Division, Synchrotron Radiation Research Organization and Institute of Molecular and Cellular Biosciences, The University of Tokyo, Bunkyo-ku, Tokyo, 113-0032 Japan; 7 Department of Cell Biology and Anatomy, Graduate School of Medicine, The University of Tokyo, Bunkyo-ku, Tokyo, 113-0033 Japan; 8 Division of Structural Medicine and Anatomy, Kobe University Graduate School of Medicine, Kobe, Hyogo, 650-0017 Japan

## Abstract

Microtubule associated protein Collapsin response mediator protein 2 (CRMP2) regulates neuronal polarity in developing neurons through interactions with tubulins or microtubules. However, how CRMP2 promotes axonal formation by affecting microtubule behavior remains unknown. This study aimed to obtain the structural basis for CRMP2–tubulin/microtubule interaction in the course of axonogenesis. The X-ray structural studies indicated that the main interface to the soluble tubulin-dimer is the last helix H19 of CRMP2 that is distinct from the known C-terminal tail-mediated interaction with assembled microtubules. *In vitro* structural and functional studies also suggested that the H19-mediated interaction promoted the rapid formation of GTP-state microtubules directly, which is an important feature of the axon. Consistently, the H19 mutants disturbed axon elongation in chick neurons, and failed to authorize the structural features for axonal microtubules in *Caenorhabditis elegans*. Thus, CRMP2 induces effective axonal microtubule formation through H19-mediated interactions with a soluble tubulin-dimer allowing axonogenesis to proceed.

## Introduction

During neuronal development, asymmetric microtubule alignment or the structural polymorphism of microtubules is essential for axon specification among many neurite processes, leading to polarized protein sorting driven by molecular motors in mature neurons^1–3^. Axonal microtubules in mature neurons are generally aligned with their plus-ends toward the cell periphery and are preferentially stained by the anti–GTP-tubulin antibody hMB11^4–6^. The abundance of GTP-tubulin in axonal microtubules (GTP-state microtubules) compared with GDP-state microtubules causes selective localization of microtubule-associated proteins (MAPs) such as molecular motor kinesin-1 (KIF5; conventional kinesin), enabling polarized axonal vesicular transport^5^. Therefore, dense alignments of GTP-state microtubules in axons help to identify the axon among many neuronal processes.

However, GTP-state microtubules are unstable *in vitro* because GTP-tubulin is hydrolyzed to GDP-tubulin immediately after GTP-tubulin is incorporated into microtubule protofilaments^7, 8^. Even when using a GTP-analog guanylyl 5′-α,β-methylenediphosphonate (GMPCPP) to mimic GTP-state microtubule polymerization *in vitro*, GMPCPP-microtubules form heterogeneous structures where GTP-tubulins, GDP-tubulins, and their intermediates might be mixed^9^. Thus, additional factor(s) such as interaction of MAPs or post-translational modifications might be required to induce and maintain axonal microtubules in neurons.

The requirements to induce axonal GTP-state microtubules *in vitro* were determined by studying a MAP called collapsin response mediator protein 2 (CRMP2/UNC33/Ulip2/CRMP-62/TOAD-64/DRP-2)^1, 10^. The founding CRMP protein, UNC-33 affects axon guidance and elongation in *C*. *elegans* ^11–13^. In addition, UNC-33 is required for axon specification by regulating axonal microtubules^13^. Kinesin motors use UNC-33-dependent microtubules to transport axon specific cargos such as synaptic vesicles. The best-characterized vertebrate CRMP2 also promotes axonal specification in cultured hippocampal neurons^14^ by direct binding of CRMP2 to tubulin-hetero-dimers^15, 16^. However, described characteristics of the interface between CRMP2 and tubulin-hetero-dimers as well as reports that CRMP2 does not bind to tubulin heterodimers but rather assembles microtubules *in vitro* to stabilize the microtubules are controversial^17, 18^.

Here we visualized how CRMP2 promotes and stabilizes axonal GTP-state microtubules using structural and functional analyses from the atomic level to the cellular level. Our data collectively suggested that CRMP2 uses two distinct binding modes: (i) binding to soluble GTP-tubulin dimers to promote polymerization of GTP-state microtubules; and (ii) binding to polymerized GTP-state microtubules to stabilize them^17^. In mode (i), the N-terminal globular domain, especially the helix H19, directly interacts with the plus-end of β-tubulin to form a compact CRMP2-tubulin hetero-trimeric complex which might be incorporated from the plus-end of the microtubule. In mode (ii), its C-terminal flexible tail tethers the wall of maturated microtubule lattices similar to classical MAPs^19^. CRMP2 uses these two binding modes at different stages of neuronal development to produce an asymmetric cytoskeleton.

## Results

### CRMP2 promotes longer GTP-state microtubule polymerization *in vitro*

**Figure 1:**
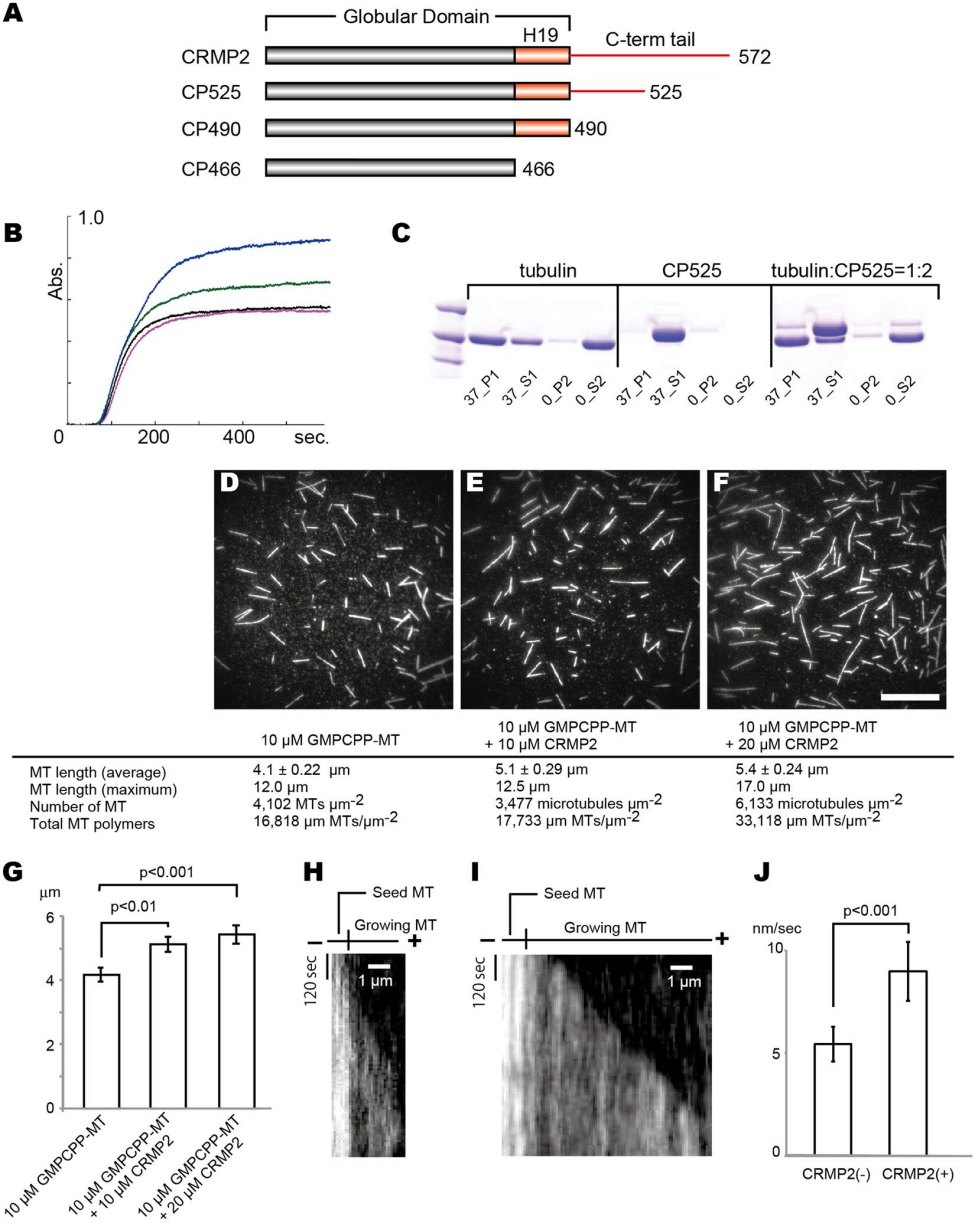
CRMP2 promotes GTP-state microtubule polymerization *in vitro*. (**A**) CRMP2 deletion constructs used in this study. (**B**) Microtubule turbidity assay in the absence/presence of CP525. Black, 20 µM GMPCPP-microtubule; magenta, 20 µM GMPCPP-microtubule and 20 µM CP525; green, 20 µM GMPCPP-microtubule and 30 µM CP525; blue, 20 µM GMPCPP-microtubule and 40 µM CP525. (**C**) Microtubule depolymerization test. Protein fractions from one cycle of CRMP2-induced reversible polymerization were separated by SDS-PAGE. Fractions in warmed pellet and supernatant, 37_P1 and 37_S1; fractions in cooled pellet and supernatant, 0_P1 and 0_S1. The results of 20 µM tubulin only, 40 µM CP525 only, and their mixture are shown. (**D–F**) Microtubule polymerization *in vitro* observed an hour after polymerization at 37 °C. Scale bar, 20 μm. (**D**) 10 µM GMPCPP-microtubules without CP525. (**E**) 10 µM GMPCPP-microtubules in the presence of 10 µM CP525. (**F**) 10 µM GMPCPP-microtubules in the presence of 20 µM CP525. (**G**) Difference of microtubule length in the absence/presence of CP525. Error bars represent the S.D. (n = 157 without CP525, n = 89 with 10 μM CP525, n = 105 with 20 μM CP525). t-test. (**H**,**I**) Kymograph of microtubule growth from the plus-end without CP525 (**H**) and in the presence of CP525 (**I**). (**J**) Microtubule growth rate without CP525 (n = 7) and in the presence of Cp525 (n = 11). Error bars represent the S.D. t-test.

To elucidate CRMP2 function *in vitro* regarding how CRMP2 affects the polymerization of GTP-state microtubules mimicked by GMPCPP-microtubules, turbidity in real time was first monitored by measuring changing absorbance at 350 nm during GTP-state microtubule polymerization in the presence or the absence of CRMP2. Of note, the latter half of the C-terminal flexible region, the C-terminal tail, is easily cleaved *in vitro*. Thus, the CP525 (amino acids T13-K525) construct including the N-terminal half of the C-terminal tail instead of full-length CRMP2 was used for *in vitro* experiments unless otherwise noted (Fig. 1A).

CP525 increased absorbance at 350 nm in a dose-dependent manner. A 1:1 molar ratio of CP525 over tubulin-dimer did not significantly affect absorbance compared with the absence of CRMP2 (Fig. 1B). A 2:1 molar ratio increased absorbance >1.5-fold than in the absence of CP525. The increased absorbance was not due to aggregation because the polymerized microtubules were disassembled into soluble tubulin-dimers after the cold treatment (Fig. 1C). Thus, high concentrations of CP525 likely stimulated polymerization of GTP-state microtubules *in vitro*. To further check whether increased absorbance actually reflected microtubule polymerization and how it was promoted in the presence of CP525, polymerized microtubules were directly visualized by fluorescently labeled tubulin-dimers after the 60 min incubations at 37 °C. A 1:1 molar ratio of CP525 over tubulin-dimer significantly lengthened GTP-state microtubules compared with in the absence of CP525 (Fig. 1D,E,G). However, total microtubule polymers were unaffected, reflecting the result of turbidity assays that showed no significant difference (Fig. 1B). At a molar ratio of 2:1, both the length of microtubules and the number of microtubules were increased. Two-fold more total microtubule polymers were polymerized in the presence of CP525 compared with in the absence of CP525 (Fig. 1D,F,G). Considering that the start time of increased absorbance in the turbidity assay was not affected by CP525, CP525 elongates and stabilizes GTP-state microtubules *in vitro* to increase the length and the number of microtubule filaments.

We further observed the effect of CP525 for the microtubule growth rate by total-internal-reflection fluorescence (TIRF) microscopy^20^. When 5 μM GTP-tubulin was perfused into the chamber in the absence of CP525, GTP-microtubules were observed to grow slowly from the GMPCPP seeds at the rate of 5.4 ± 0.84 nm/sec (Fig. 1H,J). We then perfused 5 μM GTP-tubulin in the presence of 10 μM CP525, resulting in the significant increase of the microtubule growth rate to 9.0 ± 1.5 nm/sec (Fig. 1I,J). Thus, CP525 not only elongates the GTP-state microtubules, but also accelerates the GTP-state microtubule growth rate *in vitro*.

### CRMP2 induces effective microtubule polymerization via its globular domain

As described above, CP525 which does not possess the latter half of the C-terminal tail induced an effective microtubule growth *in vitro*. That is, lack of the half of the C-terminal tail did not lose the function for the microtubule growth. Thus we next clarify the role of the C-terminal tail for this function. Using COS7 cells, the effects of the full-length CRMP2 and a series of C-terminus deletion CRMP2 constructs on the microtubule growth were compared (Fig. 1A). Most full-length CRMP2 diffused into COS7 cell cytoplasm as reported^15^. After cell membrane permeabilization by saponin, its localization along the length of microtubules was clearly visualized (top column of Fig. S1A). However, CRMP2 was not equally distributed along the length of microtubules, but exhibited punctate localization along microtubules. Deletion of a half-length of the C-terminal tail (i.e., CP525) partially disrupted the punctate microtubule-localization of CRMP2 (middle column of Fig. S1A). CP525 was weakly localized to microtubules or below cell edges. Complete deletion of the C-terminal tail (CP490; amino acids T13-E490) abolished CRMP2-microtubule-binding (bottom column of Fig. S1A). Therefore, CRMP2 uses the C-terminal tail to bind to the microtubule lattice.

**Figure 2:**
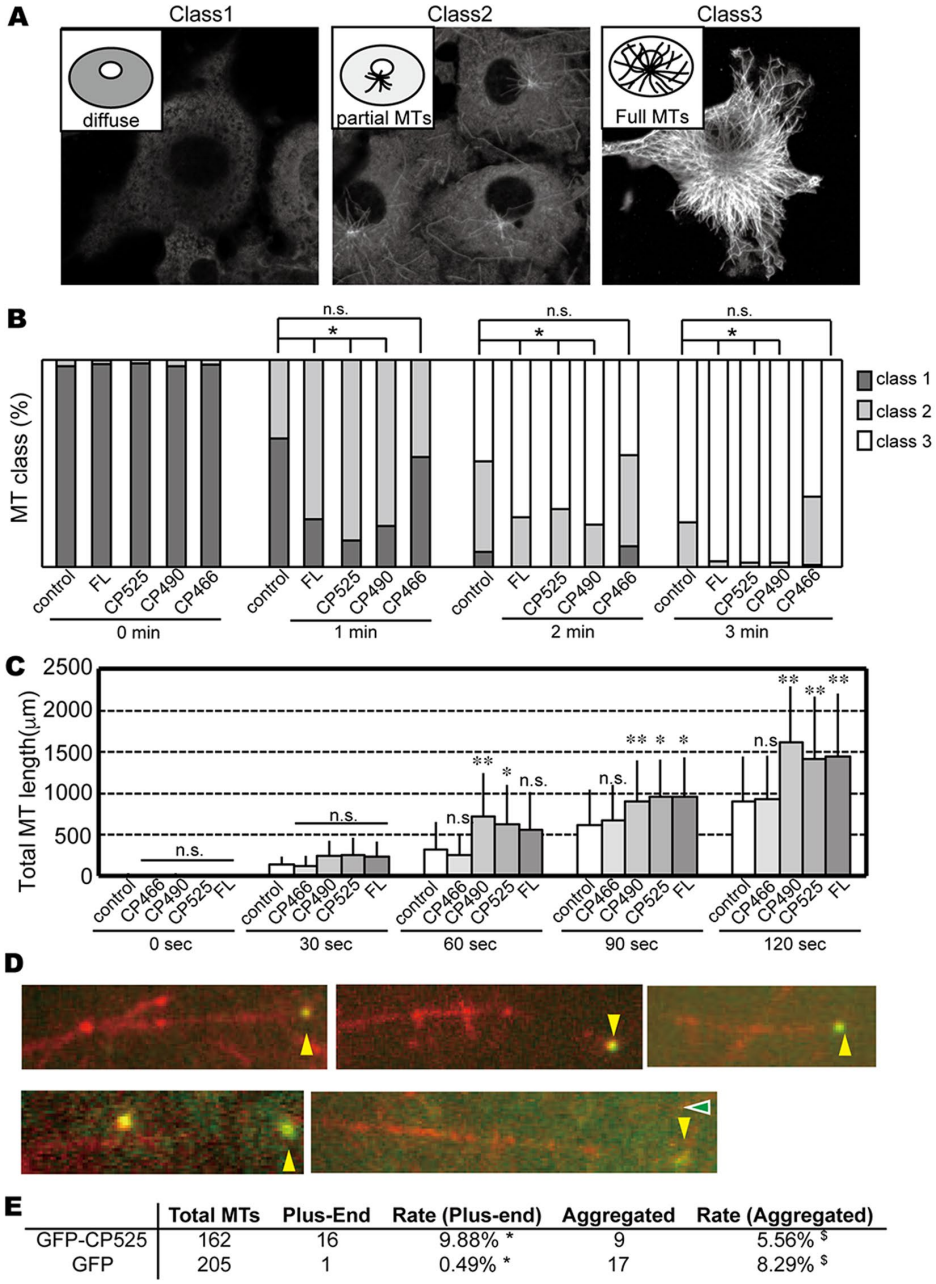
CRMP2 induces effective microtubule polymerization via its globular domain. (**A**) Classification of COS7 cells by patterns of microtubule polymerization used in the panel B. Representative images for three classes are shown. Class I (diffuse), no microtubule was observed; Class II (partial MTs), partial polymerization of microtubules was observed from the microtubule organizing center. Non-centrosomal microtubules were not found; Class III (Full MTs), microtubule polymerization was saturated. Non-centrosomal microtubules as well as centrosomal microtubules were found. (**B**) Time course of polymerization after the cold-induced microtubule depolymerization without CRMP2 or with the series of deletion constructs of CRMP2. A representative result from three independent experiments is presented. FL, full-length CRMP2. n = 100 cells for each transfection. *P < 0.05, compared to control. Chi-square test with Bonferroni correction. (**C**) Time course of microtubule elongation after the cold-induced microtubule depolymerization. The total length of microtubules in each cell was measured. Mean ± S.D. N = 23 cells. **P < 0.01, compared to control; *P < 0.05, compared to control. Dunett’s test. (**D**) GFP-CP525 localizes on the plus-end of microtubules *in vitro* (yellow arrowheads). (**E**) The localization of GFP-CP525 and GFP control. *P < 0.01; ^$^P > 0.2. t-test.

We then investigated whether microtubule polymerization induced by CRMP2 was coupled with CRMP2 localization along microtubules, and was correlated with the existence of C-terminal tail. CRMP2-transfected COS7 cells were incubated at 4 °C to depolymerize microtubules, and cells were transferred to 37 °C to initiate microtubule polymerization. Microtubules were visualized by anti-tubulin antibodies. All cells showed diffuse tubulin staining, but very few microtubules were found before the 37 °C treatment (Class 1; Fig. 2A,B). Compared with the cells without CRMP2 transfection, the recovery of microtubules was significantly faster in COS7 cells transfected with full-length CRMP2 (Fig. 2A–C). C-terminal tail deletion constructs (CP525 and CP490) also presented the similar rate of the microtubule polymerization. However, further deletion of the last helix H19 of the globular domain (CP466; amino acids 1–466) resulted in the similar rate to the COS7 cells without CRMP2 transfection, thus abolishing the effect of CRMP2 for microtubule polymerization (Fig. 2A–C). Therefore, the globular domain of CRMP2, especially the last helix H19, is required for the enhancement of microtubule polymerization by CRMP2. CRMP2-binding to the microtubule lattice through the C-terminal tail is not required to achieve this function. Hence, CP525 utilized in *in vitro* assays could serve as a good model for the stimulation of microtubule polymerization by full length CRMP2.

Of note, CRMP2 was not localized on growing microtubules but diffused into the COS7 cell cytoplasm (Fig. S1B). Therefore, the localization of CRMP2 during the microtubule growth was further investigated by TIRF microscopy *in vitro*. Unlike the classical MAPs or the typical plus-end tracking proteins like EB1, CP525 did not stay attached on the microtubules during the microtubule growth. A stable binding of CP525 to the microtubules was rarely observed in the live imaging. However, after 1% glutaraldehyde-fixation, CP525 was often observed at the plus-end of the microtubules; around 10% of the microtubule plus-ends were occupied by CP525 (Fig. 2D,E). Even though no CP525 was observed on the microtubule lattice or the microtubule minus-ends. GFP control was also rarely observed at the plus-ends of microtubules. Hence, CP525 does not surf on the plus-ends, but likely accumulates around the plus-ends to induce the GTP-state growing microtubules *in vitro*.

### CRMP2 promotes GTP-state microtubules with characteristic growing ends

**Figure 3:**
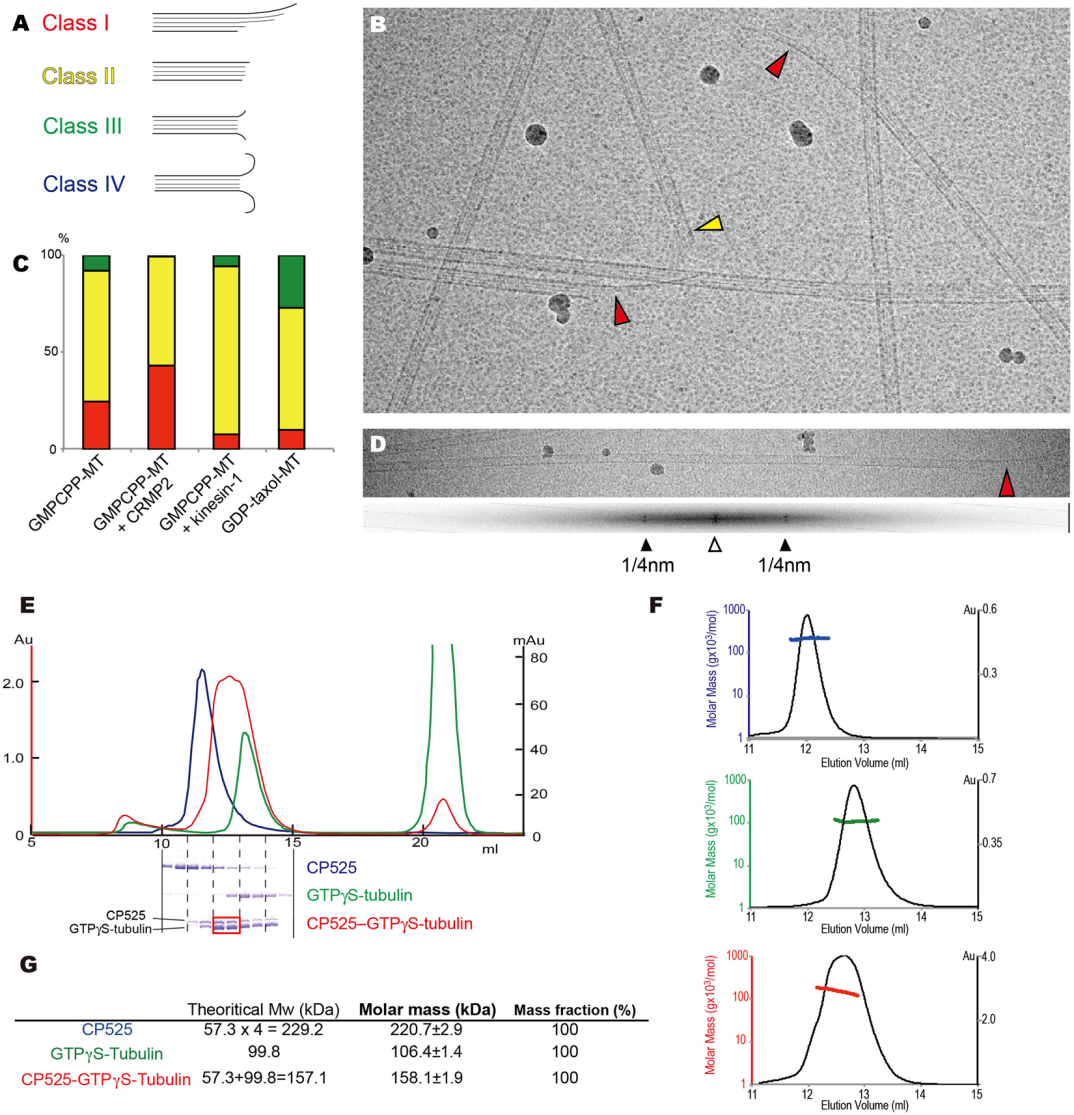
CRMP2 induces GTP-state microtubules through the interaction with soluble GTP-tubulin dimer. (**A**) Classification of microtubule ends. I, curved sheets; II, blunt ends; III, tapered ends; IV, curved ends. (**B**) Representative examples for end shapes of GMPCPP-microtubules in the presence of CP525. Red and yellow arrows show class I and class II ends, respectively. (**C**) Percentage of end types observed. GMPCPP-microtubule (n = 187); GMPCPP-microtubule+CP525 (n = 184); GMPCPP-microtubule+kinesin-1 (nucleotide free) (n = 230); GDP-taxol-microtubule (n = 205). (**D**) Cryo-EM image of CP525-induced GMPCPP microtubule (top) and its FFT image (bottom) are displayed. (**E**) Size exclusion chromatography of CP525 (blue), GTP-γS-tubulin-dimer (green), and CP525 with GTP-γS-tubulin-dimer (red) shown with SDS-PAGE results. (**F**) SEC-MALS analyses of CP525 (top), GTP-γS-tubulin (middle) and their mixture (bottom). (**G**) The molar mass determined by SEC-MALS experiments. Theoretical molecular weights calculated from the amino acid sequence were also shown for comparison.

The effect of CRMP2 on microtubule conformation was then determined by cryo-electron microscopy (cryo-EM). Because tubulin conformations in the microtubule lattice reflect the shapes of microtubule ends^21^, cryo-EM images of GMPCPP-microtubules in the presence or absence of CP525 were obtained to observe their end shapes, which were classified into four groups as previously described (Fig. 3A)^21^. Class I ends (curved sheet) are usually observed in GTP-state growing microtubules (red arrow heads in Fig. 3B), whereas class IV ends (curved end) are usually observed in depolymerized or GDP-state unstable microtubules^22^. Stable microtubules often show blunt-end conformation represented by class II or class III (yellow arrowhead in Fig. 3B).

Since the microtubule-based molecular motor kinesin-1 is one of the major MAPs that stabilize microtubules, the effects of two MAPs, CRMP-2 and kinesin-1, on microtubule-end conformation were compared. We visualized GMPCPP-microtubules in the absence of CP525, GMPCPP-microtubules polymerized with CP525, GMPCPP-microtubules complexed with nucleotide free kinesin-1, and GDP-taxol microtubules. The majority of GDP-state microtubules modified by microtubule-stabilizing reagent paclitaxel (GDP-taxol-microtubule) showed class II or III ends (Fig. 3C). The majority of GMPCPP microtubules in the absence of MAPs showed class II ends (67%), although microtubules with class I ends increased to 25%. When GMPCPP microtubules were polymerized in the presence of CP525, then, more microtubules (43%) showed class I ends (Fig. 3B–D). Considering that the minus end of microtubules usually represents a class II shape, the actual percentage of plus-end microtubules polymerized in the presence of CP525 and having class I ends is close to 86%. This rate is much higher than that observed in microtubules polymerized without CP525 (49%), indicating CP525 induces microtubules with class I sheet shapes at their plus-ends. It is thought this conformation is observed in growing GTP-state microtubules^23^. When we observed GMPCPP-microtubule decorated with another microtubule-associated protein, kinesin-1, most microtubules showed class II ends (87%), consistent with the taxol-like stabilizing effect of kinesin-1 on microtubules is observed in cryo-EM structural studies^24^.

In addition, CP525 was not likely to be localized on the microtubule lattice, consistent with the TIRF and COS7 experiments (Figs 2D, S1B), at least in a periodic manner seen for kinesin-1. Many homogeneous particles, expected to be 230 kDa CP525 tetramers, were observed in the background ice (Fig. 3B). The fast Fourier transform (FFT) image of GMPCPP microtubules in the presence of CP525 did not show the 8 nm layer lines expected from CP525 decoration, but only 4 nm layer lines corresponding to the tubulin-monomer repeat (Fig. 3D).

### CRMP2 interacts with a soluble GTP-tubulin dimer to make a hetero-trimer

The above *in vitro* and *in vivo* experiments clearly indicated that CRMP2 does not stably bind to microtubule lattice during promotion of microtubule assembly even in the presence of a flexible C-terminal tail but rather diffuses into the cytoplasm where soluble tubulin-dimers are located. However, the interaction between CRMP2 and soluble tubulin-dimer is controversial^14, 15, 17^. We thus re-examined the interaction between CP525 and GTP-tubulin-dimer at 4 °C *in vitro* by size-exclusion chromatography. The GTP analog GTP-γS (guanosine 5′-O-[γ-thio]triphosphate) was used in this case to mimic GTP because GMPCPP effectively stimulates the assembly of tubulin-dimers into microtubules even at 4 °C.

CP525 was eluted as the homo-tetramer as described previously (blue chromatogram in Fig. 3E)^25^, whereas GTP-tubulin was eluted as the hetero-dimer (green chromatogram in Fig. 3E). A 1:1 mixture of each 1 mM CP525 and GTP-tubulin-dimer then shifted the CP525 fractions to a lower molecular weight and the tubulin fractions to a higher molecular weight (red chromatogram in Fig. 3E). The peak elution fractions were 31.3 μM and approximately in a 1:1 molar ratio between CP525 and tubulin-dimer. Thus, the CP525-tubulin interactions break down CP525 homo-tetramer to form a hetero-trimer consisting of CRMP2-monomer and tubulin-dimer. It should be noted that the homo-tetramer of CP525 is stable even at the concentration ten times lower than the elution concentration of CP525-tubulin complex in the size exclusion chromatography. Thus, the dissociation constant between CP525 and tubulin is expected to be higher than the CP525–CP525 interaction in the CP525 homo-tetramer in the absence of tubulin. Nevertheless, tubulin is able to break down the CP525 tetramer, suggesting the ability of tubulin to initiate the conformational change of CP525 that decreases the affinity between CP525s in the CP525 tetramer.

The hetero-trimer formation of CP525 and tubulin-dimer was further checked by the size-exclusion chromatography combined with multi-angle light scattering (SEC-MALS) (Fig. 3F,G). As expected, the molar mass of CP525 was calculated as 220.7 ± 2.9 kDa, consistent with a homo-tetramer formation. That of GTP-γS tubulin-dimer was also 106.4 ± 1.4 kDa that is close to the theoretical molecular weight of tubulin-dimer. A 1:1 mixture of CP525 and GTP-γS tubulin-dimer then resulted in 158.1 ± 1.9 kDa calculated as the molecular weight. Considering that the tubulin-dimer is very stable in solution, this value is approximately equal to the sum of the theoretical molecular weights for the CP525 monomer and GTP-γS tubulin-dimer (157.1 kDa). This supports that CP525 and GTP-γS tubulin-dimer would form a hetero-trimer complex in solution.

### Crystal structure of CRMP2 elucidates the H19–C-terminal tail conformation

**Figure 4:**
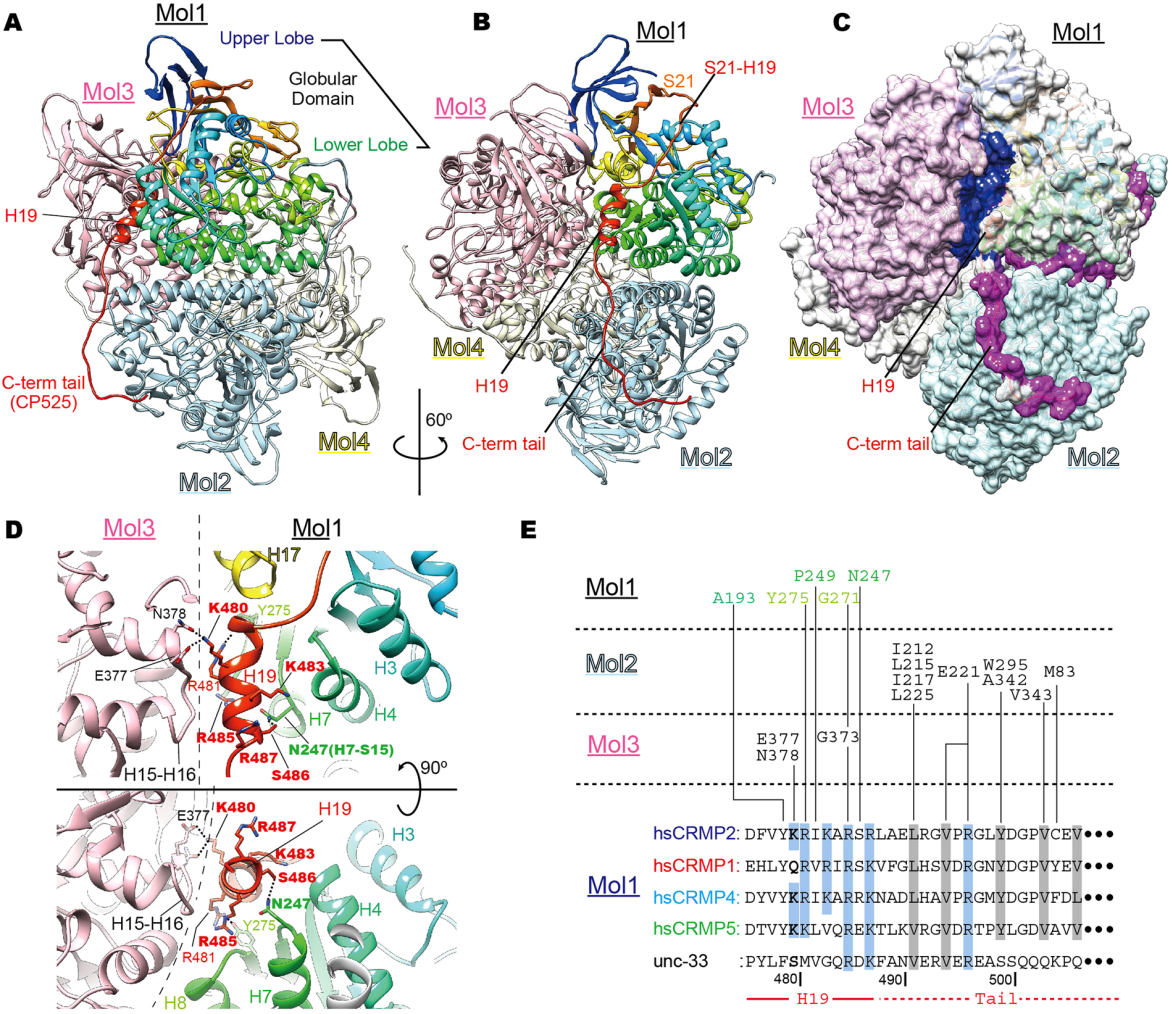
Crystal structure of CP525 elucidates the H19–C-terminal tail conformation. (**A**) Crystal structure of CP525 homo-tetramer. One monomer (Mol1) is highlighted in rainbow colors: colder colors from N-terminus and warmer colors to C-terminus. (**B**) CP525 structure seen from the left side of panel A to highlight the H19-C-terminal tail conformation. (**C**) Surface representation of CP525 structure from the same orientation with (**B**). Blue area, the interface between Mol1 and Mol3; purple area, the interface between Mol1 and Mol2. (**D**) Close-up view of H19 that serves as the interface between molecules 1 and 3 is shown. (**E**) Sequence alignment of H19 and C-terminal tail are shown with the interacting residues in the CRMP2 tetramer. The Structural detail of the C-terminal tail is described in Fig. S3. Basic residues in H19, blue background; hydrophobic residues in C-terminal tail, gray background.

We then crystallized CP525 that included half of the C-terminal tail. CP525 took homo-tetramer (Mol1, 2, 3, 4 in Fig. 4) and the overall conformation was similar to previously solved CRMPs structures^25–29^, although the flexible C-terminal tail was more clearly visualized in this study (Figs 4A and S2). CP525 consists of an N-terminal globular domain, –β-sheeted upper lobe (M1-G68) and α/β barrel lower lobe (G69-R487)–, followed by the unstructured C-terminal tail (L488-G572) that extends from the last helix H19 of the lower lobe (Fig. 4A). The helix H19 that is required for the enhancement of microtubule polymerization, is included in the lower lobe but is separated from the preceding S21 by a long 21-residue insertion of the S21-H19 loop (Fig. 4B).

The helix H19 of Mol1 is surrounded by several helices from Mol1 and Mol3 (Fig. 4B–D). H19-mediated interface area (570.58 Å^2^) accounts for 48% of the whole interface area (1,191.2 Å^2^) between Mol1 and Mol3 (blue area in Fig. 4C), thus serving as a major interface between them to form the homo-tetramer. H19 is characteristically rich in basic residues and conserved among CRMPs (Fig. 4E); more than half of the residues are basic (K480-R481-K483-R485-R487). K480 at the beginning of H19 makes a major contribution to the interaction between Mol1 and Mol3. K483 and R487 residues make little contribution to the intra- and inter- molecular interactions indicating they might be an interface with other binding partners outside CRMPs. At the end of H19, S486 also contributes to the intra-molecular interactions with N247 to stabilize the helix H19 and the initial portion of the C-terminal tail.

On the other hand, the C-terminal tail extended from the helix H19 serves as a major interface between Mol1 and Mol2. C-terminal tail-mediated interface area (1,309.4 Å^2^) accounts for 56% of the whole interface area (2334.4 Å^2^) between Mol1 and Mol2 (purple area in Fig. 4C). Contrary to the helix H19, the proximal region of the C-terminal tail is rich in hydrophobic residues; almost every three residues are conserved hydrophobic residues among CRMPs, contributing to the stabilization of the initial quarter of the C-terminal tail on the neighboring globular domain (Figs 4E and S3A). Hence, this structural study suggested that both the hydrophilic interactions between Mol1 and Mol3 through the H19 and the hydrophobic interactions between Mol1 and Mol2 through the C-terminal tail are key to stabilize the CRMP2-CRMP2 interactions in the CRMP2 homo-tetramer. In other words, the conformational changes or increasing flexibility of the H19 and/or the C-terminal tail might destabilize the homo-origomerization of CRMP2.

### Solution structure of CRMP2-GTP-tubulin complex

**Figure 5:**
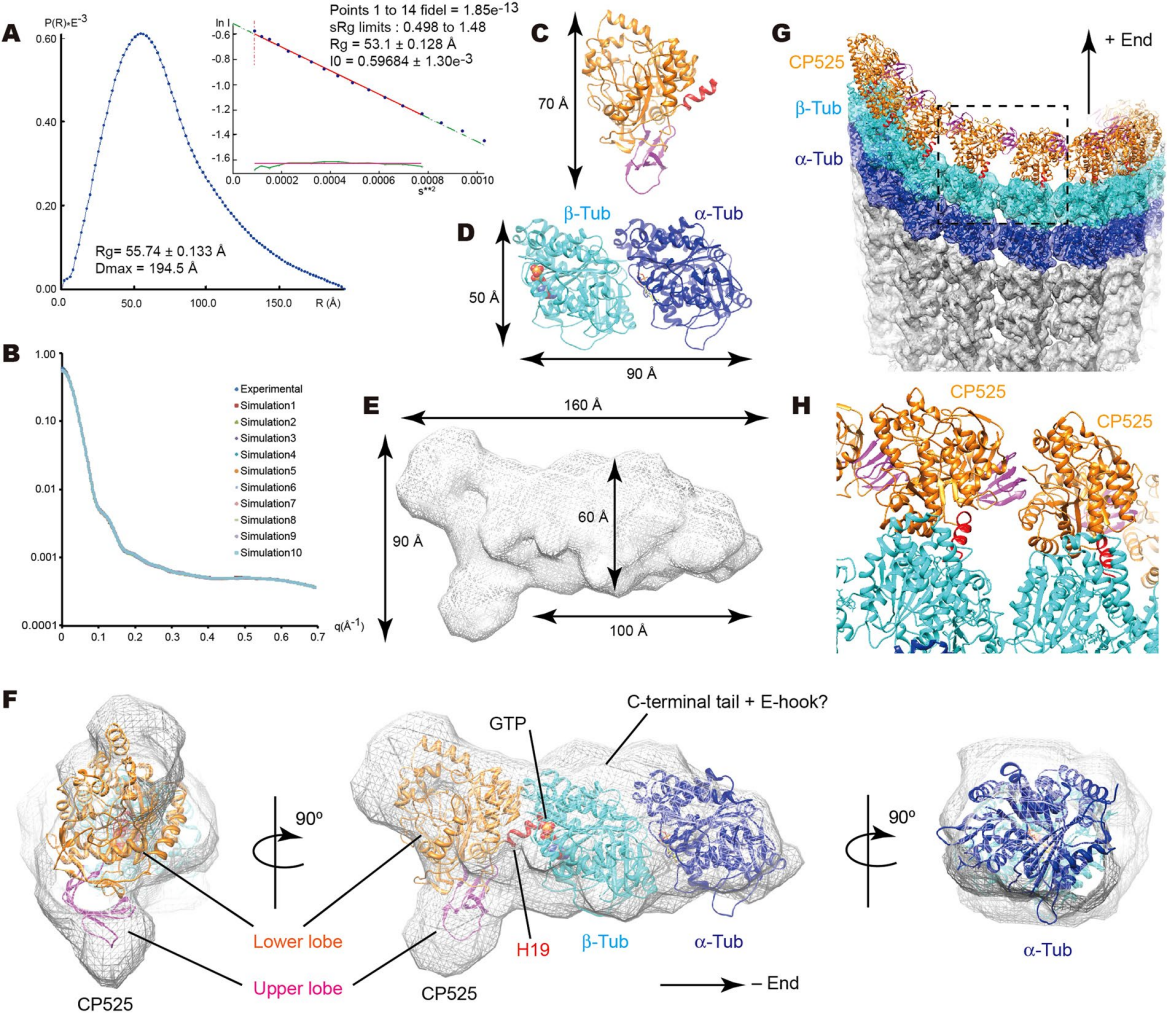
Solution structure of CRMP2-GTP-tubulin hetero-trimer complex from small angle X-ray scattering. (**A**) Pair distance distribution functions (P(r)) and the Guinier plot. (**B**) Agreement of scattering data with GASBOR models (χavg = 2.21 ± 0.082). (**C**,**D**) Ribbon models of CRMP2 solved (**C**) and tubulin-dimer (PDB ID = 1JFF) (**D**) with size indication. (**E**) SAXS envelope for the CRMP2-tubulin complex. (**F**) Fitting of atomic models of CRMP2 and tubulin-dimer into the SAXS envelope. (**G**) Fitting of SAXS model of CRMP2-tubulin complex into the plus-end of the microtubule with 13 protofilaments (EMD-5195). (**H**) Close-up view of the dotted square in panel (**G**) depicts no steric clashes between neighboring CRMP2s. Data are mean ± s.d.

Next, we characterized the CRMP2-tubulin interaction using small angle X-ray scattering (SAXS) analyses. For SAXS analysis, a 1:1 mixture of CP525 and GTP-γS tubulin eluted by gel filtration chromatography was used (Fig. 3E). A linearity of Guinier region certified the homogeneous distribution of target molecules, indicating that the majority of CP525 and tubulin was included in the homogeneous complex structure (Fig. 5A). The pair distance distribution function showed larger maximal dimension (D_max_ = 194.5 Å) than that expected from the radius of gyration (Rg = 55.74 ± 0.13 Å), suggesting the target complex does not take a globular shape but an elongated or ellipsoidal shape (Fig. 5A). Combined these experimental SAXS profiles with the biochemical assays described above (Fig. 3E,F), CP525 and GTP-γS tubulin forms a homogeneous hetero-trimeric complex consisting of the CRMP2 monomer and GTP-γS tubulin-dimer in solution. We then generated low resolution *ab initio* models of 3D arrangements with GASBOR to provide the molecular envelope shape for the CP525-tubulin complex^30^. Ten individual runs of GASBOR were performed, aligned, and averaged to calculate the SAXS envelope (Fig. 5B), which has a cylindrical elongated structure with approximately 60 Å diameter with one end expanded to take a characteristic banana-shape (Fig. 5E,F).

We then performed rigid-body fitting of atomic models of CP525 and tubulin-dimer into the SAXS envelope (Fig. 5C–E). The solved CP525 monomer perfectly fitted into the characteristic banana-shape at the end (Fig. 5F). The banana-like curvature determined the approximate rotation angle of CP525. The atomic model of tubulin-dimer (PDB ID = 1JFF)^31^ fit into the remaining cylindrical region (Fig. 5F). The cross correlation coefficient between the SAXS envelope and the density calculated from the fitted atomic model of CP525 and tubulin was 0.920, validating the fitting quality.

In the SAXS model, CP525 was located at the plus-end side of β-tubulin where the tubulin-dimers are rapidly added during microtubule polymerization (Fig. 5F). Helix H19 of the globular domain of CP525 faces the center of the plus-end side of β-tubulin to form a hetero-trimeric complex. This perfectly matches the previous result in which CRMP2 recognizes the nucleotide state of tubulin^18^, and the result of COS7 studies where deletion of helix H19 in CP466 totally abolished the enhancement of microtubule polymerization by CRMP2. In the current docking model, steric clashes between H19–C-terminal tail of CP525 and β-tubulin were observed, suggesting their conformational changes are required to form the CP525-tubulin complex. Since the helix H19 and C-terminal tail serve as major interfaces among the CP525 homo-tetramer, CP525-tubulin interaction that breaks the CP525-tetramer will destabilize the H19–C-terminal to fit the β-tubulin interface (Fig. 4C–E).

Because CRMP2 accumulates at the plus-end of microtubules *in vitro* (Fig. 2D,E), and is located at the tips of the growing axons where the plus-end of microtubules exist^14^, *in silico* fitting of the SAXS model of CRMP2-tubulin-dimer complex into the cryo-EM map of 13-protofilament microtubule (EMD-5195) at the plus-end was performed^32^ (Fig. 5G). No steric clashes were observed between the neighboring CRMP2-tubulin complexes on the neighboring protofilaments (Fig. 5G,H). Thus, all plus-ends of 13-protofilaments of a microtubule could be occupied by CRMP2-tubulin complexes and be well situated for adding GTP-tubulin to the plus-end of microtubules.

### Interface mutations of CRMP2 abolish the GTP-tubulin binding *in vitro*

To confirm the structural model of CRMP2-tubulin interaction, we generated two structure-guided interface mutants of CP525. The first target is the possible central interface of CP525 for tubulin, helix H19. Because it has characteristic basic residue clusters (Fig. 4D,E), triple alanine mutations were introduced to test their binding for the GTP-tubulin-dimer (CP525-RA: K483A–R485A–R487A). The other target was T246-N247 in the H7-S15 loop that stabilizes the end of helix H19 from the back (CP525-NA: T246A-N247A) (Fig. 4D,E). The latter mutation in CRMP1 was reported to suppress Sema3A-mediated axon repulsion in neurons^33^.

**Figure 6:**
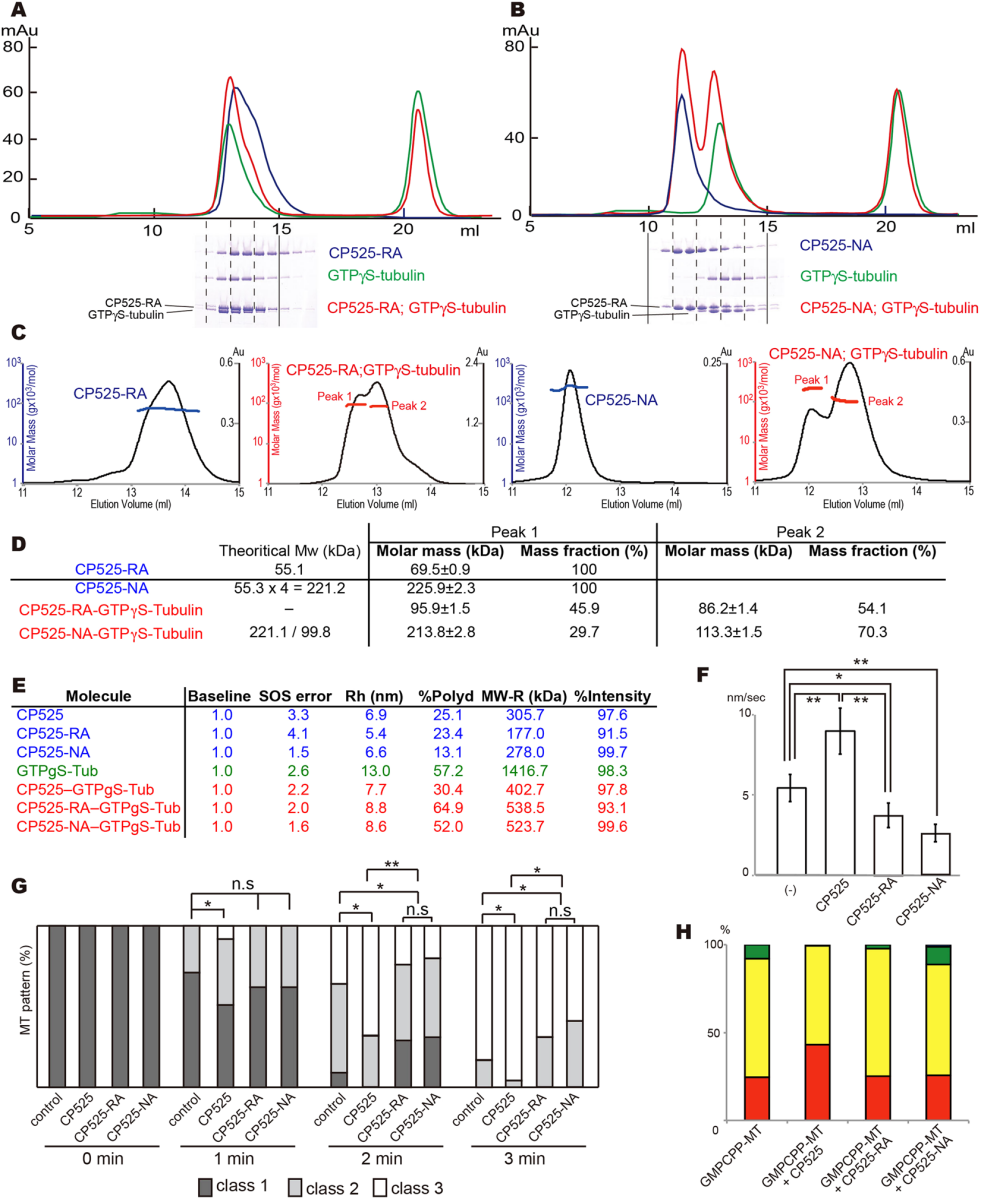
Structure-guided CP525 interface mutants abolish CRMP2-tubulin binding. (**A**,**B**) Size exclusion chromatography of CRMP2 mutants (blue), GTP-γS-tubulin-dimer (green), and CRMP2-mutants with GTP-γS-tubulin-dimer (red), shown with SDS-PAGE results. (**A**) CP525-RA. (**B**) CP525-NA. (**C**) SEC-MALS analyses of CP525 mutants and the mixtures with GTP-γS-tubulin. (**D**) The molar mass determined by SEC-MALS experiments. Theoretical molecular weights calculated from the amino acid sequence were also shown for comparison. (**E**) DLS results of CP525, CP525 mutants, GTP-γS-tubulin-dimer, and CP525/CP525 mutants with GTP-γS-tubulin-dimer. (**F**) Microtubule growth rate without CRMP2 (n = 7), in the presence of CP525 (n = 11), CP525-RA (n = 9), and CP525-NA (n = 9). Error bars represent the S.D. *P < 0.01; **P < 0.001. t-test. (**G**) Time course of microtubule polymerization after the cold-induced depolymerization without CRMP2, with CP525 wild-type or with CP525 interface mutants. A representative result from three independent experiments is shown. n = 100 cells for each transfection. *P < 0.05; **P < 0.01, compared to control. Chi-square test with Bonferroni correction. Classification is explained in Fig. 2B. (**H**) Percentage of end types observed. GMPCPP-microtubule (n = 187); GMPCPP-microtubule+CP525 (n = 184); GMPCPP-microtubule+CP525-RA (n = 200); GMPCPP-microtubule+CP525-NA (n = 198).

SEC-MALS experiments suggested CP525-RA mainly forms a mixture of homo-dimers and monomers (blue chromatogram of Fig. 6A,C,D), whereas CP525-NA forms a homo-tetramer similar to the wild type CP525 (blue chromatogram of Fig. 6B,C,D). Instability of homo-origomeric formation in CP525-RA is explained by the crystal structure; the major interface between Mol1 and Mol3 was abolished by triple alanine mutations of H19 (Fig. 4D,E). A mixture of CP525-NA and GTPγS-tubulin resulted in two peaks corresponding to the CP525-NA tetramer and tubulin-dimer, indicating that the interactions between CRMP2 and tubulin were abolished (Fig. 6B–D). In the case of a mixture of CP525-RA and GTPγS-tubulin, they were eluted in so close fractions that presence or absence of peak shifts could not be judged (red chromatogram of Fig. 6A). However, SEC-MALS experiment indicated that CP525-RA could not make the hetero-trimeric complex with tubulin-dimer; two peaks that were smaller than the tubulin-dimer but larger than the CP525-RA monomer were observed (Fig. 6C). Thus, tubulin may not interact with CP525-RA, rather a mixture of GTPγS-tubulin-dimer, CP525-RA-dimer, and CP525-RA-monomer would be existed in solution.

We further tested the interaction of CP525 mutants with GTPγS-tubulin by dynamic light scattering (DLS) performed at room temperature (Fig. 6E). Hydrodynamic radiuses (Rh) and low polydyspersities (%Polyd < 30%) of CP525 wild type and mutants are consistent with the SEC-MALS experiments. DLS from GTP-γS-tubulin represented a high polydispersity with a large hydrodynamic radius, indicating the oligomerization of tubulin in solution. A 1:1 mixture of CP525 wild type and tubulin significantly decreased polydispersity and hydrodynamic radius compared with those from tubulin only, consistent with a homogeneous compact complex formation in solution. On the other hand, both the mixture of CP525-RA and tubulin and the mixture of CP525-NA and tubulin represented similar values, high polydispersities with large hydrodynamic radiuses, indicating that tubulin did not interact or interacted very weakly with both mutants. These biochemical studies collectively represented that both interface mutations of CRMP2 abolished or severely weaken the GTP-tubulin binding ability.

We also visualized the conformational change of H19 and C-terminal tail in the crystal structure of CP525-NA (Table S1 and Fig. S3B). As expected, N247A broke the hydrogen bond with S486 of H19 (Fig. S3C). Because K480-mediated inter-molecular interactions at the beginning of H19 did not change in CP525-NA, H19 rotates around the start of H19 so the end of H19 becomes approximately 1.0 Å more distant from the H7-S15 loop than that of wild type. It further induced a small conformational change of the initial quarter of the C-terminal tail that might lead to a larger conformational change of the remaining C-terminal tail. The NA mutation causes conformational changes of H19 and the C-terminal tail, thus destabilizing the tubulin-binding site of CRMP2 to decrease its binding ability for tubulin. These experiments collectively suggested the importance of T246-N247 for the stabilization of the interface helix H19 of CRMP2 for tubulin.

### Two interface mutants could not promote GTP-state microtubule polymerization

SEC-MALS experiments indicated that both CP525 interface mutants did not form the hetero-trimeric complex with tubulin-dimer. We then observed the effect of the interface mutations on microtubule growth rate by TIRF microscopy. As already described, the growth rates of GTP-microtubules were 5.4 ± 0.84 and 9.0 ± 1.5 nm/sec, in the absence and the presence of the wild type CP525, respectively (Fig. 1H–J). In the presence of 10 μM interface mutants of CP525, however, the significant decrease of the microtubule growth rate even comparing to that without CP525 were observed (3.8 ± 1.0 nm/sec with CP525-RA and 2.6 ± 0.4 nm/sec with CP525-NA) (Fig. 6F). Thus, the interface mutants of CRMP2 could not promote, but rather slow down the polymerization rate of GTP-state microtubules.

We also investigated whether the enhancement of microtubule polymerization by CP525 was affected by the interface mutations in COS7 cells. Microtubule polymerization in COS7 cells was observed after cold-induced microtubule depolymerization. As described above, the enhancement of microtubule polymerization was significantly faster in COS7 cells transfected with wild type CP525 than those without CRMP2 (Fig. 6G). In the cells with the transfection of the interface mutants (CP525-RA or CP525-NA), however, microtubule polymerization was significantly slower than those with wild type CP525, and was slower even comparing to those without CRMP2, consistent with the TIRF experiments (Fig. 6F).

We further observed by cryo-EM whether the interface mutations affect the conformation of microtubule ends or not (Fig. 6H). The existence of the wild type CP525 increased the population of microtubules with class I ends, as described above. In the presence of the interface mutants CP525-RA or CP525-NA, however, the majority of GMPCPP microtubules showed class II ends (CP525-RA, 73%; CP525-NA, 63%), whereas the class I ends decreased to 25% in both mutants (Fig. 6H). This distribution was similar to that without CRMP2. These mutant experiments collectively indicated that CRMP2-tubulin interaction through the proposed interface not only promotes the microtubule growth rate, but also characterizes the polymerized microtubule in the axon specific form.

### Two interface mutants disturb axon elongation in chick

**Figure 7:**
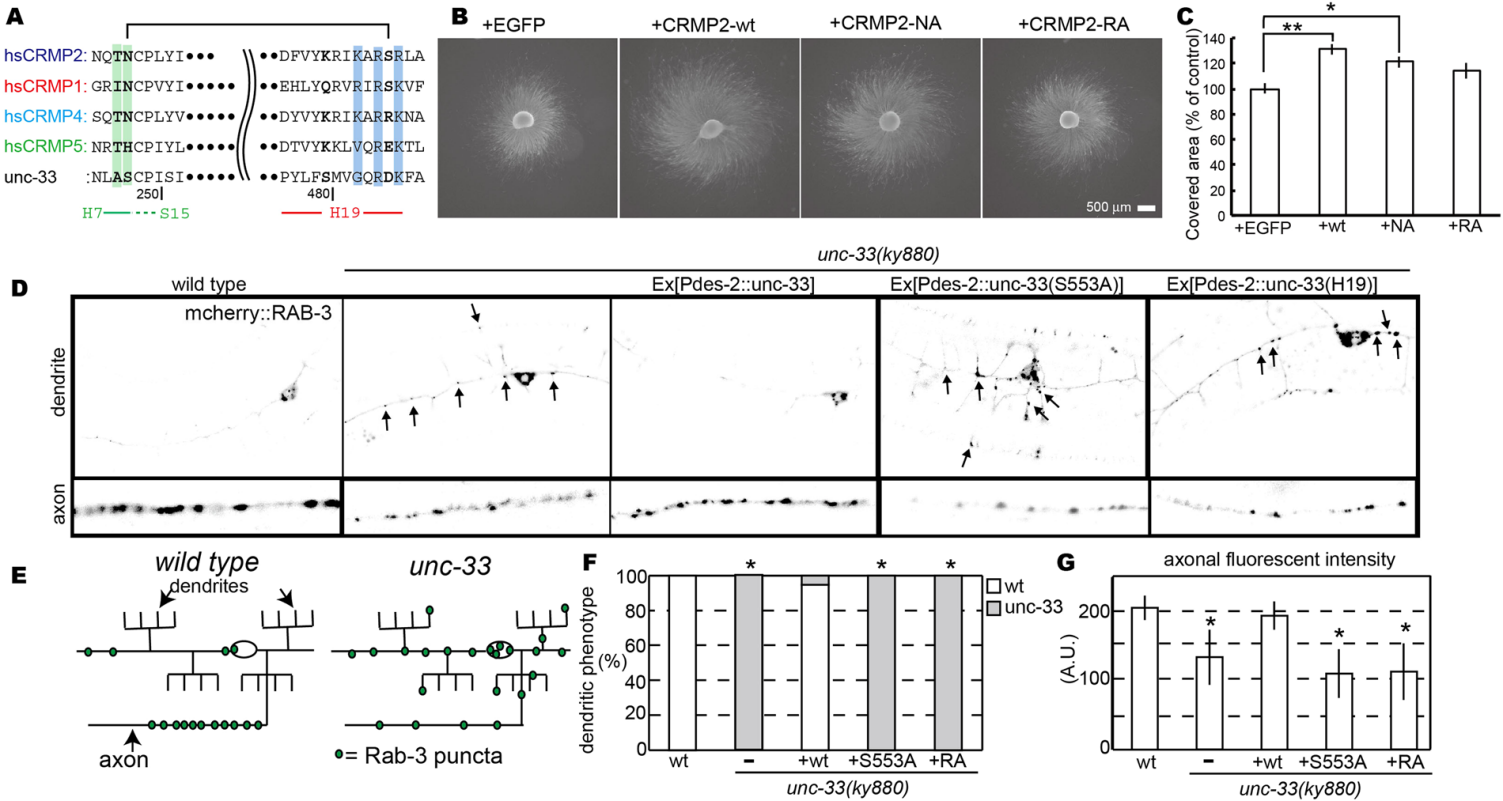
Two interface mutants affect CRMP2 functions in chick and *c-elegans* neurons. (**A**) Sequence alignment of the regions to be mutated. T246-N247, green; K483–R485–R487, blue. N247 hydrogen-bonds with S486 in the wild type CP525 structure. (**B**) Micrograph of DRG explants. Chick E7 DRG explants were infected with recombinant herpes-simplex virus harboring the indicted constructs (see Fig. S4 for infection efficiency). The neurites were visualized by α-tubulin immunostaining. Note that CRMP2-wild type (wt) enhances the neurite-outgrowth from the explant. This effect is canceled by the expression of RA mutant. Scale bar, 500 µm. (**C**) Scored graph. The covered area with the neurites was measured with ImageJ software. The relative area size of each sample was scored against the average of EGFP controls in the same experiment. Bar graph represents the mean ± s.e.m of 24 samples in each condition from 4 independent experiments. *P < 0.05; **P < 0.01, one-way ANOVA with post-hoc Tukey-Kramer test. (**D–G**) UNC-33 and UNC-33 mutants were expressed in *unc-33*(*ky880*): kyIs445 and the localization of mCherry::RAB-3 was observed in PVD neurons. (**D**) The localization of synaptic vesicles visualized by mCherry::RAB-3 in PVD neurons. wt, *unc-33*(*ky880*) and *unc-33*(*ky880*) expressing UNC-33 mutants were observed in the dendritic region and the axonal region. (**E**) Schema showing the localization of synaptic vesicles in wt and unc-33. (**F**) Dendritic mislocalization was statistically analyzed. The phenotype was sorted to either wt or unc-33 and counted. *P < 0.01, compared to wt, Chi-square test, n = 100 worms. (**G**) The axonal fluorescent intensity was measured in each strain. Mean ± S.D., *P < 0.01, compared to control, Dunnett test, n = 20 worms.

We finally go back to the importance of the association of CRMP2/UNC-33 with tubulin *in vivo*. From the *in vitro* structural and biochemical analyses, the helix H19 serves as the interface between CRMP2/UNC-33 and tubulin (Fig. 5F). Helix H19 is supported by association with the H7-S15 loop (Figs 4C,D and 7A). The NA (T246A-N247A in the H7-S15 loop) and RA (K483A–R485A–R487A in the helix H19) mutations abolish the CRMP2 function to polymerize GTP-state microtubules. Thus, the effects of these mutations for axon elongation were observed in chick neurons. The effect of CRMP2-overexpression on neurite outgrowth in chick dorsal root ganglia (DRG) neurons were examined because the transient expression of CRMP2-wild type in rat hippocampal neurons enhanced the outgrowth^34^. Using a herpes-simplex virus system, we introduced EGFP-CRMP2-wild type, NA and RA mutants into chick E7 DRG neurons (Fig. 7B). As expected, overexpression of CRMP2-wild type significantly enhanced neurite-outgrowth in DRG neurons. Overexpression of NA mutant also promoted outgrowth compared with the negative control, although it tends to show less enhancement than the wild type. The overexpression of the RA mutant then did not show any enhancement or dominant-negative effects on neurite-outgrowth (Fig. 7B,C). Based on the structural and biochemical studies of interface mutants, these mutations decreased the affinity for soluble tubulin, thus the overexpression of mutants over intrinsic CRMP2 would not affect intrinsic CRMP2-tubulin interactions. Thus, overexpression of the NA or the RA mutants showed a milder phenotype against neurite-outgrowth.

### CRMP2-tubulin interactions are required for axon-specific cargo sorting in *C*. *elegans*

We investigated the effects of NA and RA mutations on the establishment of axon-dendrite polarity in *C*. *elegans* neurons. *unc-33* is a *C*. *elegans* homologue of CRMP2^11–13^. Previous studies showed *unc-33* establishes the neuronal polarity by promoting the formation of axonal microtubules used by kinesin to transport axonal cargos^13^. Synaptic vesicles are mislocalized to dendrites in *unc-33* mutants. Synaptic vesicles were visualized with mCherry::RAB-3 in PVD neurons in wild type and *unc-33*(*ky880*) as described previously^13^. Synaptic vesicles were specifically localized to the ventral PVD axon in wild type (Fig. 7D,E). In *unc-33*(*ky880*) synaptic vesicles were mislocalized to the dendrite (Fig. 7D,E), consistent with a previous report^13^. Strong dendritic mislocalization was observed in *unc-33*(*ky880*) (Fig. 7F) and the axonal fluorescent signal was significantly weaker than wild type in *unc-33*(*ky880*) (Fig. 7G). The expression of wild type UNC-33 in the PVD neuron reduced the mislocalization of synaptic vesicles in dendrites and restored axonal fluorescent signals (Fig. 7D,F and G). Two UNC-33 mutants were generated. In *C*. *elegans* UNC-33, A552-S553 in the H7-S15 loop and G786-R788-K790 in H19 correspond to T246-N247 and K483–R485–R487, respectively (Fig. 7A). S553A and G786A-R788A-K790A mutations were generated for the NA and RA mutations. S553A mutation was predicted to destabilize the association between the H7-S15 loop and helix H19 because S553 is expected to interact with D789 in H19, while the RA mutation was predicted to disrupt binding to tubulin. The UNC-33 mutants were expressed in PVD neurons in unc-33 mutants, but did not rescue the mutant phenotype of dendritic mislocalization or axonal fluorescent intensity (Fig. 7D,F and G). Thus, the H19-corresponding domain of unc-33 is essential for the establishment of neuronal polarity. Considering that the helix H19 serves as an interface for tubulin and its mutation abolishes the binding affinity to tubulin *in vitro*, the data suggest that the association between helix H19 and tubulin is required for the establishment of neuronal polarity *in vivo*.

## Discussion

We performed a series of structural and functional analyses *in vitro*, using COS7 cells and neurons, and clarified how CRMP2 bind to tubulin dimer and microtubules. The interaction between CRMP2 and soluble tubulin-dimer was reported previously^14, 15^. However, a recent BIACORE experiment failed to show an interaction between them^17^. Indeed, most residues in the minimal-binding fragment of CRMP2 for tubulin (amino acids 323–381) identified by Fukata *et al*. are buried in the globular domain when CRMP2 forms a tetramer. The biochemical data and the pseudo-atomic structure of the CRMP2-tubulin complex shown here might help explain these controversies. Moreover, our data gives an insight how GTP microtubules, an important axonal polarity cue, are induced by CRMP2 in the axonogenesis.

**Figure 8:**
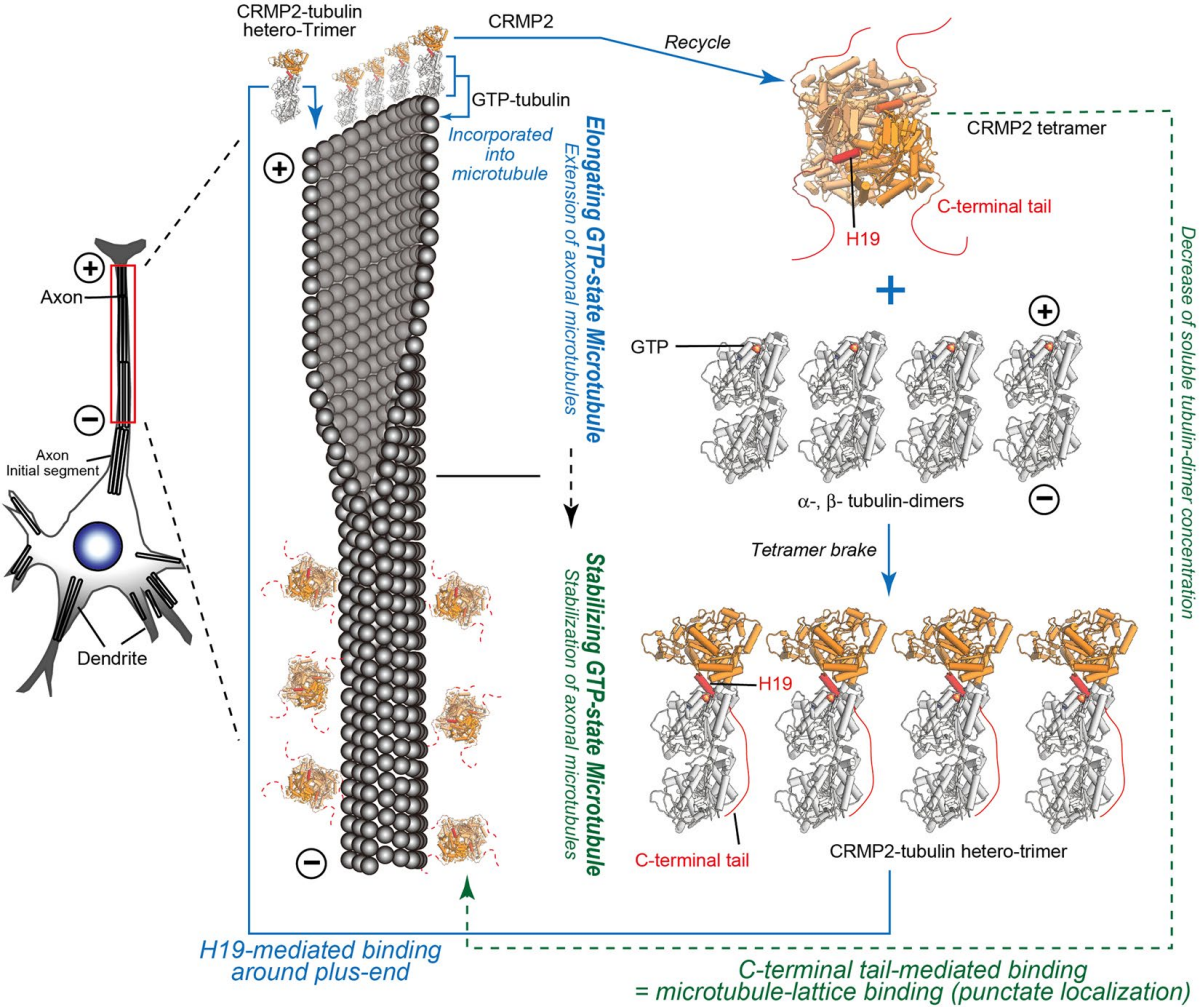
Structural model for the double functions of CRMP2: elongating GTP-state microtubules and stabilizing GTP-state microtubules. See the main text for details.

We summarize our research results in Fig. 8. (1) CRMP2 forms a homo-tetramer and helix H19, rich in basic residues, is a major interface for homo-tetrameric formation. The C-terminal tail extending from H19 is exposed on the surface and also contributes to homo-tetrameric formation. (2) Full-length CRMP2 is present along the length of maturated microtubules, tethered by its C-terminal tail, consistent with previous results^17^. (3) CRMP2 effectively polymerizes GTP-state microtubules with characteristic sheeted plus-ends at high rates, via direct interaction between CRMP2 and soluble GTP-tubulin. CRMP2 does not present on the microtubule lattice during this process, but accumulates around the plus-ends of microtubules. (4) Soluble GTP-tubulin hetero-dimer breaks the CRMP2 homo-tetramer to form a hetero-trimer of CRMP2 and GTP-tubulin-dimer. The helix H19 of CRMP2 serves as a central interface that faces the plus-end side of β-tubulin. In this conformation, CRMP2 caps the plus-end of GTP-tubulin, and therefore the plus-end of GTP-state microtubules. (5) The interface mutants of CRMP2 for GTP-tubulin consistently suppressed the growing GTP-state microtubule formation *in vitro* or the axon specific microtubule formation in *C*. *elegans*. Thus, CRMP2-tubulin interactions make a major contribution to axon specification in developing neurons through the effective elongation of GTP-state microtubules.

These *in vitro* structural-based findings indicated the molecular mechanisms of CRMP2 function, especially in axonal microtubule formation in developing neurons. During axonogenesis, CRMP2 is highly enriched in growing axons^1, 14, 15^. In cultured hippocampal neurons, CRMP2 is concentrated in the distal part of the expected axon at stage 3^1, 35^, when microtubules are selectively stained by GTP-tubulin antibody^5^. Kinesin-1 preferentially binds to GTP-microtubules to transport axon-specific cargo^5^. Our data suggest that during this stage, CRMP2 forms a hetero-trimer with GTP-tubulin-dimer at the tip of expected axons to induce GTP-state microtubule polymerization. These microtubules are “authorized” to develop specific characteristics in the growing microtubules, and are highly stained by GTP-tubulin antibody. During this initial stage, CRMP2 is not present on the microtubule lattice, but interacts with GTP-tubulins that will be incorporated into the plus-end of the axonal microtubules. Thus, CRMP2 accumulates around the plus-end of the microtubules, apparently similar to +TIPs like EB1 or XMAP215. Helix H19 in the globular domain functions as the main interface for GTP-tubulin to form a hetero-trimer (Fig. 5F). Indeed, Helix H19 is required for the establishment of axon-dendrite polarity in *C*. *elegans* neuron (Fig. 7). However, unlike most +TIPs which bind to the outer surface of microtubule ends or tubulins, the interface of GTP-tubulin is the plus-end side of the β-tubulin that is normally shielded by longitudinal contacts in the polymerized microtubules. Therefore, the rapid dissociation of CRMP2 from the plus-end is required after the incorporation of the tubulin-dimer to the microtubule. After detaching from the microtubule, CRMP2 will be immediately recycled to bind to the next tubulin-dimers. Rapid imaging of CRMP2 turnover at the growing microtubule ends would be required to test this hypothesis^36^.

Our proposed model in which the H19-mediated CRMP2-tubulin interaction promotes axonal formation could explain most of the unc-33 mutations that affect microtubule polarity in worm neurons^13^. Both *unc-33*(*mn407*) and *unc-33*(*e1193*) do not have the lower lobe or are expected to have misfolded one. *unc-33*(*ky869*) does not have both the upper and lower lobes. *unc-33*(*ky880*) destabilizes preceding β-sheet of H19, thus expected to alter the H19 position or the conformation. These mutants that exhibit severe phenotypic effect for the microtubule polarity all show the drastic conformational change of H19 or the absence H19, lending credit to our proposed model. On the other hand, *unc-33*(*e204*) that has the mutation at the junction of upper and lower lobes represents mildest phenotype among them. In this mutant, the relationship between upper lobe and lower lobe are expected to alter, suggesting that some modulation by upper lobe may exist for the function of H19-mediated axonal formation. Further studies are necessary to clarify the role of the upper lobe.

In mature axons (stage 5), CRMP2 presents along the length of the microtubule to stabilize GTP-state microtubules similar to the function of classical MAPs^17^. CRMP2-microtubule binding in this phase is mediated by the C-terminal tail. The C-terminal tail mediated interactions with microtubules may not break the CRMP2 homo-tetramer because the plus-end side of β-tubulin is shielded in the polymerized microtubules. Therefore, CRMP2 homo-tetramers might tether on microtubules via the flexible C-terminal tail (Fig. 8), in which CRMP2 diffuses along the microtubule similar to the weak-binding state of molecular motor kinesin^37^. This binding character might reflect the punctate localization of CRMP2 along the microtubules (Fig. S1A).

Thus, our structural and functional analyses revealed a unique property of CRMP2 as a MAP. Although other MAPs often only have a single interface with microtubules, CRMP2 uses different interfaces for soluble tubulin-dimers and/or assembled microtubules. Helix H19 binds to soluble tubulin-dimers and is used when GTP-state microtubules are growing. The C-terminal tail binds to GTP-state microtubules to stabilize them. The order of priorities might depend on the balance between the local concentration of CRMP2 and soluble tubulin-dimers or the decreasing rate of soluble tubulin-dimers compared with the assembled tubulin-dimer. In either case, the interaction between CRMP2 and tubulins/microtubules produces two distinct functions using two interfaces in a different time or location. It is an unprecedented and interesting example not only of interactions between MAPs and microtubules, but of protein-protein interactions in general.

## Methods

### Preparation of CRMP2 and GMPCPP-tubulin for *in vitro* experiments

The CRMP2 constructs CP525 [7 × His-tag and human CRMP2 residues 1–525] and CP490 [7 × His-tag and human CRMP2 residues 1–490] were cloned into pET21b (*Novagen*) *and* expressed in *Escherichia coli BL21* (*DE3*) *cells* (*Novagen*). Constructs were purified by immobilized metal affinity chromatography using complete His-Tag Purification Resin (Roche). Proteins were purified further by size exclusion chromatography (AKTA Explorer 10 S, HiLoad 16/60 superdex200 column; GE Healthcare). Elution fractions were concentrated to >100 mg/ml using an Amicon Ultra-15 Centrifugal Filter Unit (Millipore). Tubulin was purified from porcine brains by six cycles of polymerization and depolymerisation as described previously^38^. GMPCPP-tubulin was prepared as described previously^9^.

### Polymerization of GMPCPP microtubules in the presence of CRMP2

Ten μM GMPCPP-tubulin was incubated in polymerization buffer (PEM (80 mM Pipes-KOH, pH 6.8, 1 mM EGTA, 1 mM MgCl_2_), 1 mM GMPCPP, and 6% DMSO) at 4 °C for 30 min in the presence/absence of 10.0, 15.0 or 20.0 μM CP525 and then clarified by centrifugation at 4 °C for 30 min at 100,000 × g using a rotor (himac CS120GX; Hitachi Koki) in an ultracentrifuge (S55A2; Hitachi Koki). Supernatant was polymerized at 37 °C for 60 min, and microtubules were collected by centrifugation through a 20% glycerol cushion at 37 °C for 10 min at 25,000 × g using a rotor (himac CS120GX; Hitachi Koki) in an ultracentrifuge (S55A2; Hitachi Koki).

### Microtubule turbidity assay

Twenty μM GMPCPP-tubulin was mixed on ice with 0, 20, 30, or 40 μM CP525 in PEM buffer and 200 μl of each reaction mixture was used. Microtubule polymerization reaction was started simultaneously by a temperature shift to 37 °C and monitored by FP-777Win spectrofluorometer (JASCO) at 350 nm.

### Microtubule polymerization assay

Microtubule growth was observed as described previously with slight modifications^20^. Porcine brain tubulin was purified and tetramethylrhodamine (TMR) labeled. GMPCPP microtubule seeds and Polarity-marked microtubules were made as described^39, 40^. Nucleotide free KIF1A was immobilized on a coverslip using PentaHis antibody (Qiagen,RRID: AB_2619735). The surface of the coverslip was further coated with casein (Wako Chemical). After washing, TMR-labeled GMPCPP-microtubule seeds (unlabeled tubulin:TMR-tubulin = 10:1) were injected into the flow chamber and washed out using PEM buffer supplemented with oxygen-scavenger. Then, 5 μM TMR-labeled soluble tubulins (unlabeled tubulin:TMR-tubulin = 30:1) with or without 10 μM unlabeled CRMP2 were injected just before observation. Time-lapse observation was performed at 37 °C using the ELYRA P.1 system (Carl Zeiss) in the TIRF mode. The data were collected every 5 to 10 seconds. Microtubule growth was analyzed using kymographs generated with the “Multiple Kymograph” plug-in for ImageJ made by J. Rietdorf (FMI Basel) and A. Seitz (EMBL Heidelberg).

To identify the CRMP2 localization during MT growth, 5 μM TMR-labeled soluble tubulins (unlabeled tubulin:TMR-tubulin = 30:1) with 10 μM GFP-CRMP2 were injected and incubated at 37 °C for 10 min. Then PEM buffer with 1% glutaraldehyde were injected into the chamber and observed.

### Grid preparation and cryo-EM image acquisition

A 5-µl drop of polymerized microtubules (3–10 μM) with CRMP2 (3–10 μM) was placed onto a glow-discharged holey carbon grid (R2/2, Quantifoil). After 30 sec, the solution was wicked away with a piece of Whatman no.1 After 60 sec, the grid was plunge-frozen into liquid ethane by using a semi-automated vitrification device (Vitrobot Mark IV, FEI) with 5 sec in 100% humidity at 27 °C. Data acquisition was performed by using 200-kV field emission cryo-EM (Tecnai Arctica, FEI) at 53,000-fold nominal magnification. All data were collected with a total electron dose of 15 e^−1^/Å^2^ at a pixel size of 1.95 Å/pixel.

### COS-7 experiments

COS-7 cells were cultured in a 5% CO2 incubator at 37 °C. Dulbecco’s modified eagle medium (DMEM) (Nacalai tesque) was supplemented with 10% bovine serum as culture medium. For observation, cells were transferred to glass bottom dishes (Iwaki or MatTek corporation).

To observe the localization of CRMP2 and CRMP2 mutants, 14 hours after transfer to glass bottom dishes, plasmids were transfected by Lipofectamine 2000 (Life Technologies). Cells were treated with PEM supplemented with 10% glycerol and 0.01% saponin, and fixed with 4% paraformaldehyde. Cells were washed with PBS twice and incubated with 1/1000 anti-tubulin antibody DM1A (Sigma) for 1 hour. Cells were washed with PBS twice and incubated with 1/100 Alexa568-labelled secondary antibody (Life Technologies) for 30 min. After three washes with PBS, cells were observed.

To monitor microtubule polymerization in COS cells, mutant plasmids were transfected as described above. Twenty-four hours after transfection, culture medium was changed to 50 μl of DMEM with 10% bovine serum. Then, cells were transferred to a 4 °C incubator and incubated for 2 hours. Then, cells were transferred on a 37 °C degree hot plate. At the indicated time point, cells were fixed with PBS supplemented with 4% paraformaldehyde and 20 μM paclitaxel (Sigma). Then, microtubules were visualized as described above.

### SEC-MALS analysis

Protein samples were loaded onto a SEC column (Bio-Rad ENrich 650) at a flow rate of 0.5 mL min^−1^ in SEC. 30 buffer (20 mM Hepes-KOH pH 7.4, 30 mM NaCl, 1 mM MgCl_2_) at room temperature using the HPLC system Prominence (Shimadzu). The column was inline with a UV-absorbance detector SPD-20A at 280 nm (Shimadzu) and a DAWN 8+ MALS detector (Wyatt Technology, 658 nm laser). Molar masses were calculated using ASTRA (Wyatt Technology) with the dn/dc value of 0.185 mL g^−1^. Bovine serum albumin was used as the calibration standard.

### Crystallization, X-ray data collection, and structure determination

Crystals of CP525, CP490, and CP525-NA were grown by the vapor diffusion method at 20 °C. Each 2-μl protein solution at 10–16 mg/ml was mixed with 2-μl of reservoir buffers (RB). RB for CP525 contained 15% PEG8000, 0.1 M Tris pH 8.5, and 100 mM DTT. RB for CP490 contained 1.5 M ammonium sulfate and 0.1 M Tris pH 7.0. RB for CP525-NA contained 11% PEG6000, 0.1 M tri-sodium citrate pH 5.6, and 0.1 M Lithium sulfate. X-ray diffraction data were collected at 100 K at BL41XU (CP525), BL32XU (CP490), and BL26B2 (CP525-NA) of SPring-8. All data were indexed, integrated and processed using HKL2000^41^. The structures were solved by molecular replacement using PHASER^42^. Atomic coordinate 2VM8 was used as a starting model^27^. Several rounds of iterative model building and refinement were performed using COOT^43^ and PHENIX^44^. The final crystallographic models of CP525, CP490, and CP525-NA were solved at 1.8 Å, 2.1 Å, and 2.2 Å resolutions with *R*_*work*_ */R*_*free*_ values of 0.187/0.214, 0.177/0.202, and 0.159/0.190 (Table S1).

### Small angle X-ray scattering data acquisition analysis and modeling

SAXS data for reconstructions of the CP525–GTPγS-tubulin complex were collected using BioSAXS-1000 (RIGAKU). SAXS was measured at concentrations of 5.0, 7.5, and 10.0 mg/ml at room temperature. SAXS data were processed by components of the ATSAS package^45^. Scattering curves were normalized and merged together in PRIMUS. The radius of gyration (*R*_*g*_) was initially calculated from the Guinier plot. The pair distance distribution function (P(r)) was calculated using the program package GNOM. The value for *D*_max_ was determined by testing the quality of the fit to the experimental data and 194.5 Å was used in the *ab initio* modeling. *Ab initio* modeling was performed with GASBOR^30^. Dummy atom models resulting from ten individual GASBOR runs were aligned and averaged using DAMAVER. A molecular envelope was generated from the filtered averaged bead model using the *pdb2vol* component of the *Situs* package^46^. Atomic models of tubulin (1JFF)^31^ and CP525 solved here were rigidly fit into the SAXS envelope manually, followed by local adjustment using the fit-in-map tool in UCSF Chimera^47^.

### Plasmid and recombinant herpes simplex virus construction

Coding fragments of EGFP and CRMP2 (wild type, CPFL-NA, and CPFL-RA) fusion constructs were PCR-amplified with two primers: EGFP-1f (5′-atcgcggccgcaccATGGTGAGCAAGGGCGAGGAGCTG-3′) and hCRMP2-endr (5′-atctctagaTTAGCCCAGGCTGGTGATGTTGGCACG-3′). These fragments were digested with *Not*I and *Xba*I and cloned into a pHSV vector. Generated plasmids were transfected into 2–2 cells and subsequently infected with IE2 defective herpes-simplex virus (5dl1.2). Control virus was prepared with the pHSV vector containing the EGFP coding region.

### DRG culture and neurite outgrowth assay

Twenty-four-well tissue culture plates (Greiner) were coated with 50 µg/ml poly-L-lysine and 8 µg/ml mouse laminin. DRG were dissected from E7 or E8 chick embryos and placed in a well containing 500 µl of F12 Ham’s medium supplemented with 2% fetal bovine serum, 16.7 ng/ml 2.5 S NGF, 50 U/ml penicillin, and 50 µg/ml streptomycin. Explants were infected with recombinant herpes-simplex viruses harboring the EGFP-CRMP2-wild type or mutants. Negative controls were infected with HSV expressing EGFP. Explants were cultured at 37 °C for one day. More than 50% of neurites showed EGFP-fluorescence. Neurites were immunostained with anti-α-tubulin rat monoclonal antibodies and Alexa594-conjugated anti-rat IgG. Entire images of neurites from each DRG explant were captured through ×2 objective lens equipped with an Olympus IX-70 microscope and a Spot-2e CCD (Spot) camera.

### *C*. *elegans* experiments

*C*. *elegans* was maintained using standard procedures^48^. *unc-33*(*ky880*)*; kyIs445* and Pdes-2::unc-33L were described previously^13^. Mutations were introduced to the Pdes-2::unc-33L plasmid by PCR-based mutagenesis with KOD-plus high fidelity DNA polymerase (TOYOBO, Tokyo, Japan). Injections were performed as described^49^. In short, wild type and mutant unc-33 plasmids (10 ng/μl) and Podr-1::DsRed (50 ng/μl) were injected to *unc-33*(*ky880*); *KyIs445* mutant worms. DsRed positive worms were transferred to new plates and stable transmission of the extrachromosomal array was determined. For rescue experiments, only DsRed positive worms were observed and analyzed by LSM710 confocal microscope (Carl Zeiss).

For the statistical analysis of the dendritic phenotype, worms were observed in a blind fashion. The observer did not know genotypes until the end of the test. Each worm was classified as “*wild type*” or “*unc-33*” by the synaptic vesicle localization. 100 worms were observed for each genotype and plotted as bar graphs. For the quantification of axonal fluorescent intensity, worms were observed in the same manner as the dendritic analysis. The mean fluorescent intensity was measured using Image J (NIH). A 100-μm region of the axon was selected randomly to measure the mean fluorescent intensity.

### Statistics

Statistics methods and the number of samples were described in the figure legends. Microsoft Excel (Microsoft) and Graphpad Prism ver.7 (Graphpad software) were used to perform statistical analysis.

### Data Availability

The coordinates for the crystal structures have been deposited in the Protein Data Bank under following accession codes. CP525, 5 × 1 A; CP490, 5 × 1 C; CP525-NA, 5 × 1D. Correspondence and requests for materials should be addressed to R.N. (ryo.nitta@riken.jp).

## Electronic supplementary material

Supplementary Information
