# Supplementary material for: Structural basis for CRMP2-induced axonal microtubule formation
Source: Sci Rep. 2017 Sep 6;7:10681. doi: 10.1038/s41598-017-11031-4 (PMC5587665; doi:10.1038/s41598-017-11031-4)
Supplement: Supplementary file 1 — Supplementary Information [file 41598_2017_11031_MOESM1_ESM.pdf]

Supplementary information

Structural basis for CRMP2-induced axonal microtubule formation

Shinsuke Niwa[1], Fumio Nakamura[2,3], Yuri Tomabechi[4], Mari Aoki[4], Hideki Shigematsu[4], Takashi Matsumoto[5], Atsushi Yamagata[6], Shuya Fukai[6], Nobutaka Hirokawa[7], Yoshio Goshima[2], Mikako Shirouzu[4], & Ryo Nitta[4,7,8]*

[1]*Frontier Research Institute for Interdisciplinary Sciences and Department of Life Sciences, Tohoku University, Aoba-ku, Sendai 980-8578, Japan*

[2]*Department of Molecular Pharmacology and Neurobiology, Yokohama City University Graduate School of Medicine, Kanazawa-ku, Yokohama 236-0004, Japan.*

[3]*Department of Biochemistry, Tokyo Women's Medical University, Shinjuku-ku, Tokyo 162-8666, Japan.*

[4]*RIKEN Center for Life Science Technologies, Tsurumi-ku, Yokohama 230-0045, Japan*

[5]*Application Laboratories, Rigaku Corporation, 3-9-12 Matsubara-Cho, Akishima, Tokyo 196-8666, Japan*

[6]*Structural Biology Laboratory, Life Science Division, Synchrotron Radiation Research Organization and Institute of Molecular and Cellular Biosciences, The University of Tokyo, Bunkyo-ku, Tokyo 113-0032, Japan*

[7]*Department of Cell Biology and Anatomy, Graduate School of Medicine, The University of Tokyo, Bunkyo-ku, Tokyo 113-0033, Japan*

[8]*Division of Structural Medicine and Anatomy, Kobe University Graduate School of Medicine, Kobe, Hyogo, 650-0017, Japan*

Correspondence to: Ryo Nitta (ryo.nitta@riken.jp)

Supplementary Figures

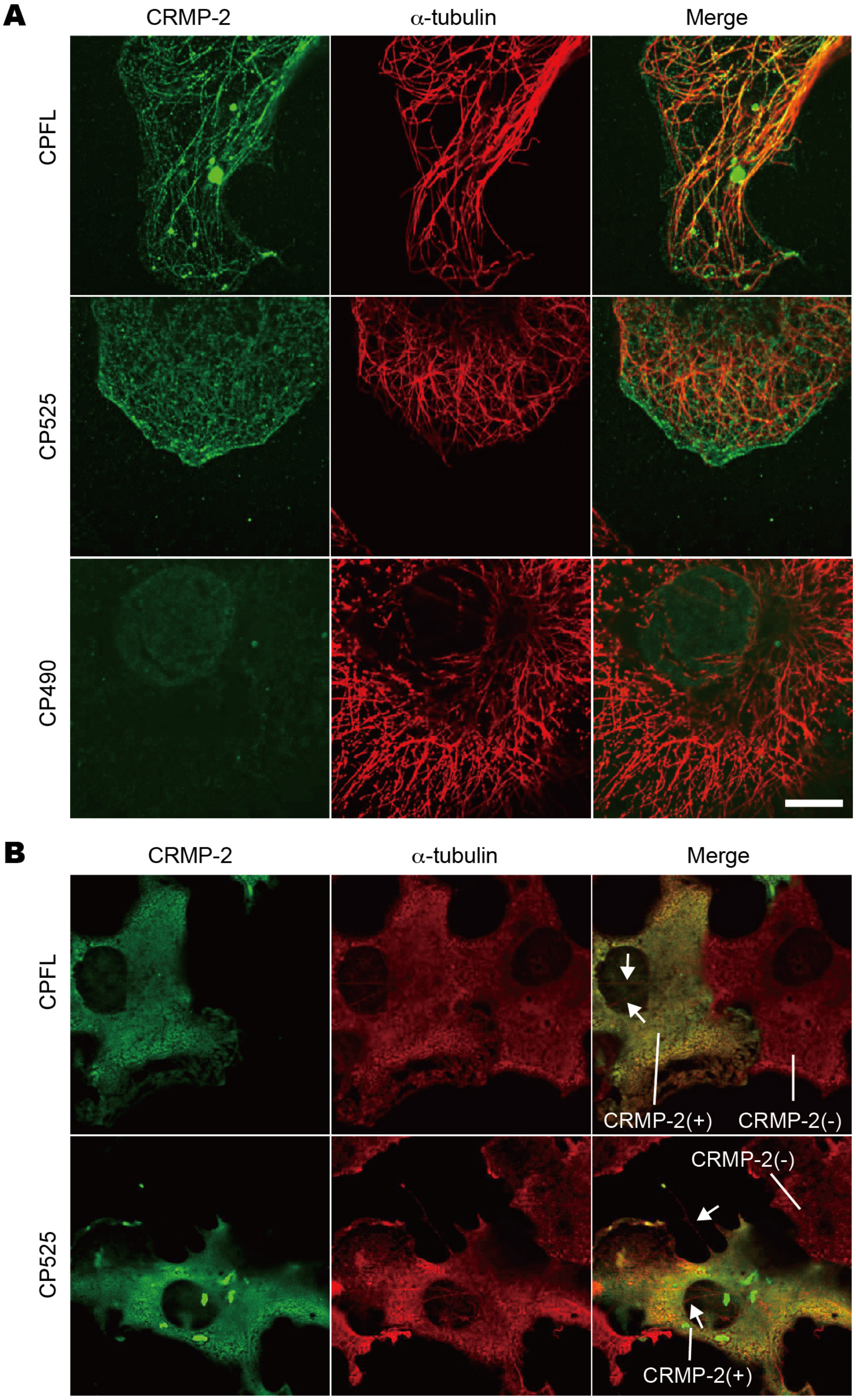

Figure S1. CRMP2 requires the C-terminal tail for its localization along microtubules. (A) Localization of CRMP2 in COS7 cells permeabilized by saponin. Scale bar, 10 μm. **(B)** CRMP2 does not localize on microtubules during the initiation of microtubule polymerization. Arrows indicate polymerized microtubules. Scale bar, 20 μm. CRMP-2(+), CRMP2-trasfected cells; CRMP-2(-), Cells without overexpression of CRMP2.

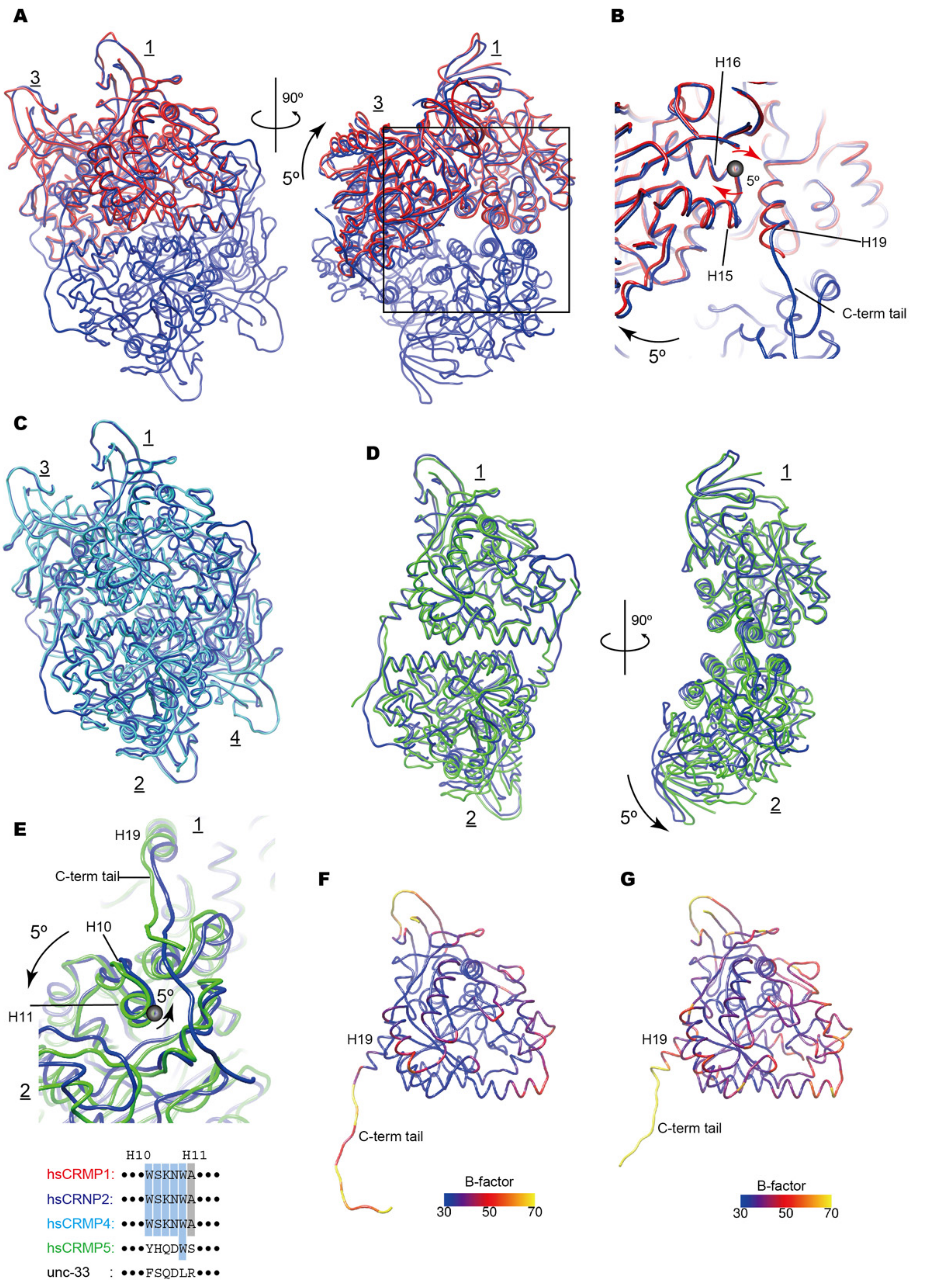

Figure S2. Structural differences among CRMPs. Red, CRMP1; blue, CRMP2; Cyan, CRMP4; green CRMP5. **(A)** Superimposed CRMP1 and CRMP2 structures. The 5° rotation of molecule 3. **(B)** Close-up view around H19 contacts indicated by a solid square in panel **(A)**. Ball indicates the rotation center of molecule 3 of CRMP1 caused by the difference of N480 in CRMP1 and K480 in CRMP2. **(C)** Superimposed CRMP2 and CRMP4 structures indicate their high similarity. **(D)** Superimposed CRMP2 and CRMP5 structures. The 5° rotation of molecule 2. **(E)** Close-up view around the C-terminal tail of molecule 1. Amino-acid sequence alignment among CRMPs. Ball indicates the rotation center of molecule 2 of CRMP5 caused by the difference of H10-H11 loop between CRMP2 and CRMP5. **(F, G)** B-factor values of CRMP2 **(F)** and CRMP5 **(G)** are indicated by colors. Stabilization of the C-terminal tail might increase the stability of the preceding helix H19 in CRMP2.

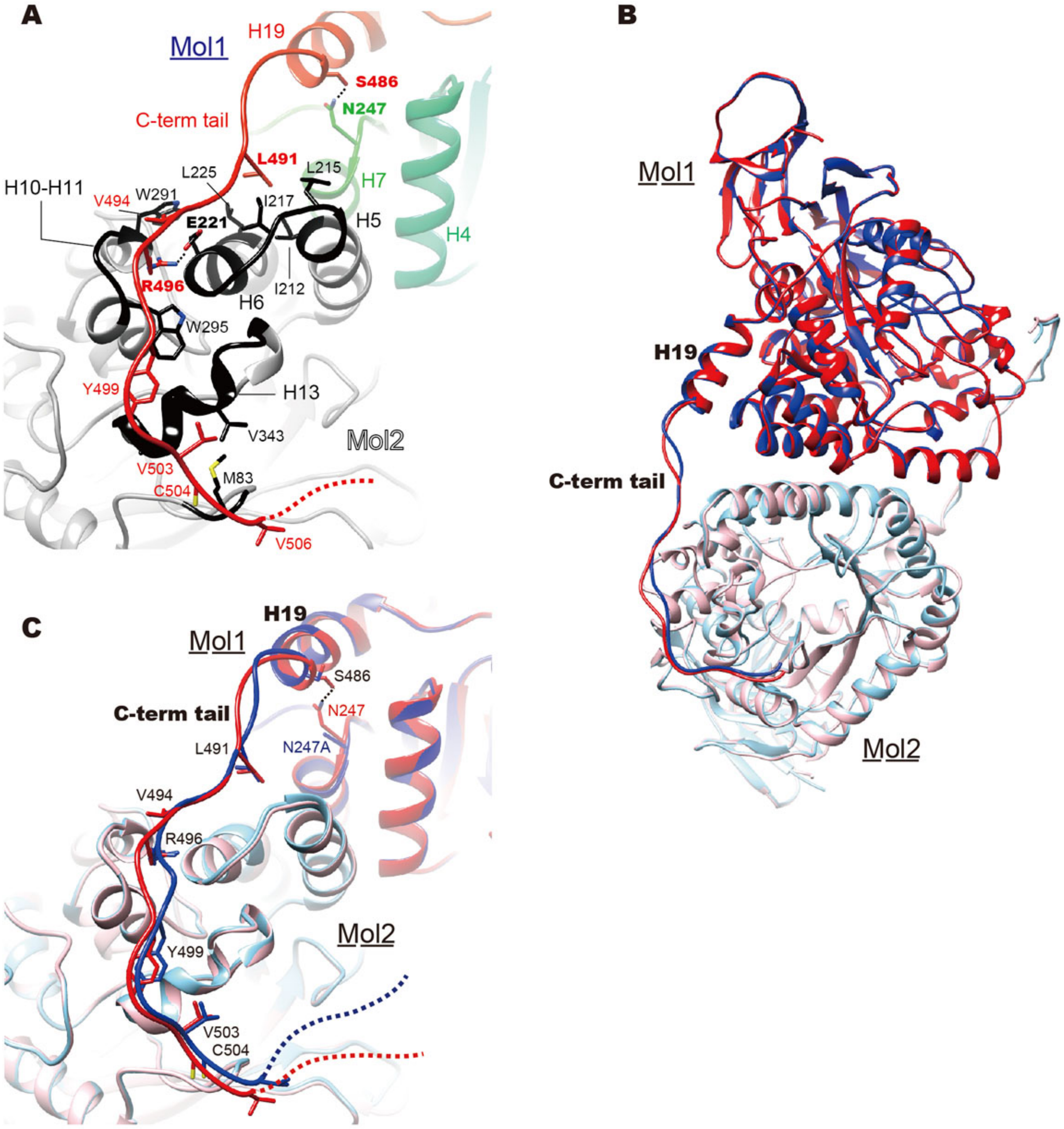

Figure S3. Structure of the C-terminal tail in CP525 wild type and CP525-NA. **(A)** The C-terminal tail was extended from helix H19, which is stabilized by a hydrogen bond between S486 and N247. The proximal region of the C-terminal tail is rich in hydrophobic residues, contributing to the stabilization of the initial quarter of the C-terminal tail on the neighboring globular domain (Fig. 4E). L491 makes first contact by insertion into the hydrophobic pocket formed by the four hydrophobic residues of neighboring molecule 2. The salt bridge between R496 of molecule 1 and E221 of molecule 2 is buried in the hydrophobic groove. These two residues are perfectly conserved among CRMPs and UNC-33 in *c-elegans*. **(B)** Crystal structure of CP525-NA (blue/light blue) superimposed on CP525 wild type (red/pink). **(C)** Close-up view of H19 and C-terminal tail of CP525-NA superimposed on CP525 wild type.

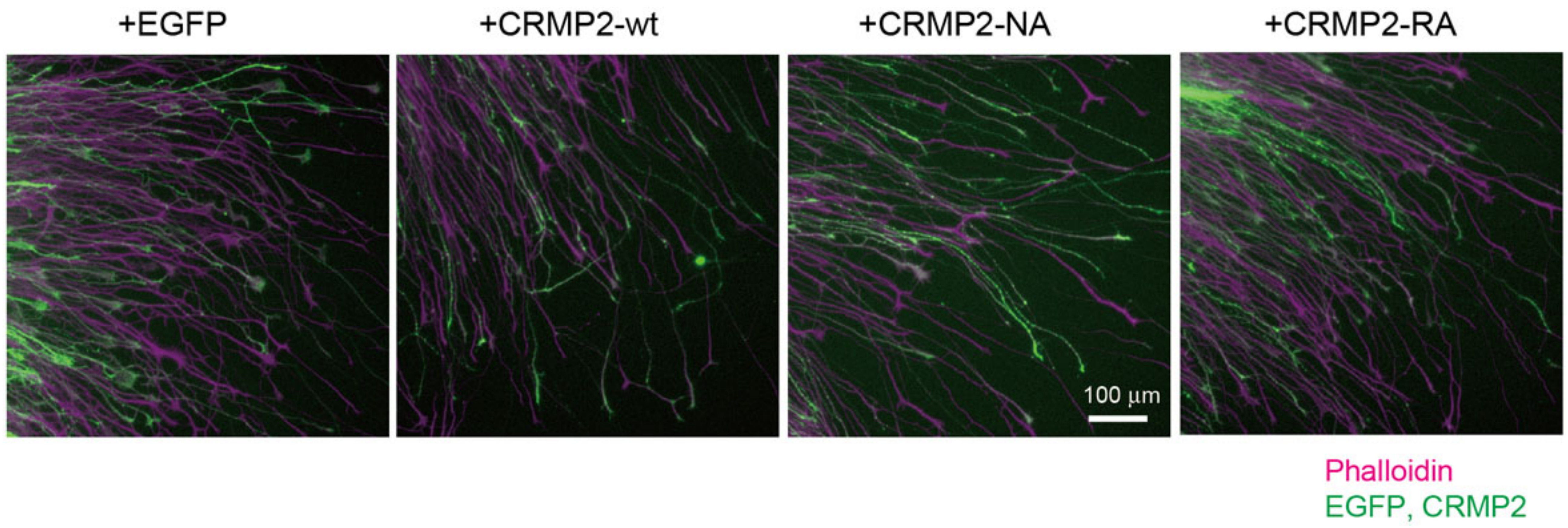

Figure S4. Infection efficiency of DRG explants. Chick E7 DRG explants were infected with recombinant herpes-simplex virus (HSV) harboring the indicted constructs. The neurites were visualized by α-tubulin immunostaining (magenta) and EGFP (green). HSV-infection efficiency is approximately 50% in each condition. Scale bar, 100 μm.

Supplementary Table

Table S1. Data Collection and Refinement Statistics

	CP525	CP490	CP525-NA
**Data collection**	BL41XU	BL32XU	BL26B2
Space group	$I4_122$	$P4_22_12$	$I4_122$
Cell dimensions			
a, *b*, *c* (Å)	114.1, 114.1,196.1	126.7, 126.7, 148.2	114.2, 114.2, 193.6
α, β, γ (°)	90.0, 90.0, 90.0	90.0, 90.0, 90.0	90.0, 90.0, 90.0
Resolution (Å)	50-1.82 (1.85-1.82)	50-2.10 (2.14-2.10)	50-2.20 (2.24-2.20)
R_{merge}	0.091 (0.997)	0.203 (0.933)	0.134 (0.933)
$I / \sigma I$	9.5 (2.5)	3.5 (2.0)	4.9 (2.7)
Completeness (%)	99.9 (100.0)	100.0 (100.0)	100.0 (100.0)
Redundancy	14.5 (11.6)	9.8 (9.4)	14.8 (14.7)
**Refinement**			
Resolution (Å)	37.3-1.82	43.3-2.10	41.5-2.20
No. reflections	57,874	70,576	32,839
R_{work} / R_{free}	0.187/0.214	0.177/0.202	0.159/0.190
No. atoms			
Protein	3,793	7,397	3,788
Ligand/ion			
Water	400	701	368
B-factors (Å^2)	33.4	34.2	30.8
R.m.s. deviations			
Bond lengths (Å)	0.008	0.009	0.003
Bond angles (°)	1.133	1.199	0.777
